# A nematode model to evaluate microdeletion phenotype expression

**DOI:** 10.1093/g3journal/jkad258

**Published:** 2023-11-13

**Authors:** Katianna R Antkowiak, Peren Coskun, Sharon T Noronha, Davide Tavella, Francesca Massi, Sean P Ryder

**Affiliations:** Department of Biochemistry and Molecular Biotechnology, University of Massachusetts Chan Medical School, Worcester, MA 01605, USA; Department of Biochemistry and Molecular Biotechnology, University of Massachusetts Chan Medical School, Worcester, MA 01605, USA; Department of Biochemistry and Molecular Biotechnology, University of Massachusetts Chan Medical School, Worcester, MA 01605, USA; Department of Biochemistry and Molecular Biotechnology, University of Massachusetts Chan Medical School, Worcester, MA 01605, USA; Department of Biochemistry and Molecular Biotechnology, University of Massachusetts Chan Medical School, Worcester, MA 01605, USA; Department of Biochemistry and Molecular Biotechnology, University of Massachusetts Chan Medical School, Worcester, MA 01605, USA

**Keywords:** microdeletion, nematode, genome instability, pleiotropy, phenotype, cytokinesis, sterility

## Abstract

Microdeletion syndromes are genetic diseases caused by multilocus chromosomal deletions too small to be detected by karyotyping. They are typified by complex pleiotropic developmental phenotypes that depend both on the extent of the deletion and variations in genetic background. Microdeletion alleles cause a wide array of consequences involving multiple pathways. How simultaneous haploinsufficiency of numerous adjacent genes leads to complex and variable pleiotropic phenotypes is not well understood. CRISPR/Cas9 genome editing has been shown to induce microdeletion-like alleles at a meaningful rate. Here, we describe a microdeletion allele in *Caenorhabditis elegans* recovered during a CRISPR/Cas9 genome editing experiment. We mapped the allele to chromosome V, balanced it with a reciprocal translocation crossover suppressor, and precisely defined the breakpoint junction. The allele simultaneously removes 32 protein-coding genes, yet animals homozygous for this mutation are viable as adults. Homozygous animals display a complex phenotype including maternal effect lethality, producing polynucleated embryos that grow into uterine tumors, vulva morphogenesis defects, body wall distensions, uncoordinated movement, and a shortened life span typified by death by bursting. Our work provides an opportunity to explore the complexity and penetrance of microdeletion phenotypes in a simple genetic model system.

## Introduction

Microdeletion syndromes are complex pleiotropic developmental diseases typically caused by multilocus deletions that are often too small to be detected by karyotyping. Microdeletion syndromes display variable penetrance depending on the extent of the deletion and the genetic background (Watson *et al.* 2014). The most common is 22q11.2DS, also known as DiGeorge or velocardiofacial syndrome (Watson *et al.* 2014; Guna *et al.* 2015; Goldenberg 2018; Motahari *et al.* 2019). This disease affects an estimated 1 in 4,000 people and manifests a wide variety of disease phenotypes, including heart abnormalities, craniofacial defects, cleft palate, short stature, psychiatric and intellectual disorders, and immunodeficiency (reviewed in (Watson *et al.* 2014; Motahari *et al.* 2019)). About 10% of cases are familial with an autosomal dominant inheritance pattern (Kelley *et al.* 1982), and the rest result from spontaneous deletions formed during gametogenesis (Carelle-Calmels *et al.* 2009). The extent and impact of the syndrome can vary widely among diagnosed patients, and some individuals are nearly asymptomatic (Sullivan 2019). Ninety percent of cases are caused by a 3 Mb deletion on chromosome 22 that disrupts approximately 46 protein-coding genes (Sullivan 2019). Current evidence suggests the disease phenotypes are caused by complex genetic interactions between multiple disrupted genes and are strongly influenced by genetic background (Carelle-Calmels *et al.* 2009; Guna *et al.* 2015; Motahari *et al.* 2019). A pressing question, not just for this disease but more generally for all microdeletion syndromes, is whether these interdependencies can be exploited to develop therapies to mitigate disease severity. More broadly, understanding how complex multigenic phenotypes manifest will help better clarify the relationship between genotype and phenotype. For example, how do variables such as loss-of-heterozygosity, genetic background, and new spontaneous lesions modify the array of phenotypes produced?

Microdeletion alleles can also arise during targeted genome editing experiments (Kosicki *et al.* 2018; Thomas *et al.* 2019). There is extraodinary interest in developing genome editing technology to treat human disease (reviewed in (Wang *et al.* 2017)). However, experiments in mouse and human cells and embryos reveal that Cas9—the most commonly used genome editing tool (Cong *et al.* 2013; Jinek *et al.* 2013; Tzur *et al.* 2013)—can cause microdeletion lesions, leading to new concerns about applying this technique to patients (Kosicki *et al.* 2018). The frequency at which such events happen is not well understood. On target “collateral damage”, as it has been termed, can be difficult to detect using PCR or short-read-based DNA sequencing technology (Thomas *et al.* 2019).

The small bacterivorous nematode *Caenorhabditis elegans* is widely used to study gene function in ageing, development, and numerous other aspects of animal physiology. The genomes of *C. elegans* and closely related species have been sequenced, and the genome size is relatively small (∼100 Mb) making whole genome resequencing cost-efficient. A large collection of mutant alleles—including large deficiencies—is widely available to researchers through the *Caenorhabditis* Genetics Center. In addition, thanks to CRISPR/Cas9, it is now routine to make targeted gene disruptions and replacements in this model organism (Chen *et al.* 2013; Chiu *et al.* 2013; Dickinson *et al.* 2013; Arribere *et al.* 2014; Ghanta, Chen, *et al.* 2021a; Ghanta, Ishidate, *et al.* 2021b).

**Fig. 1.**
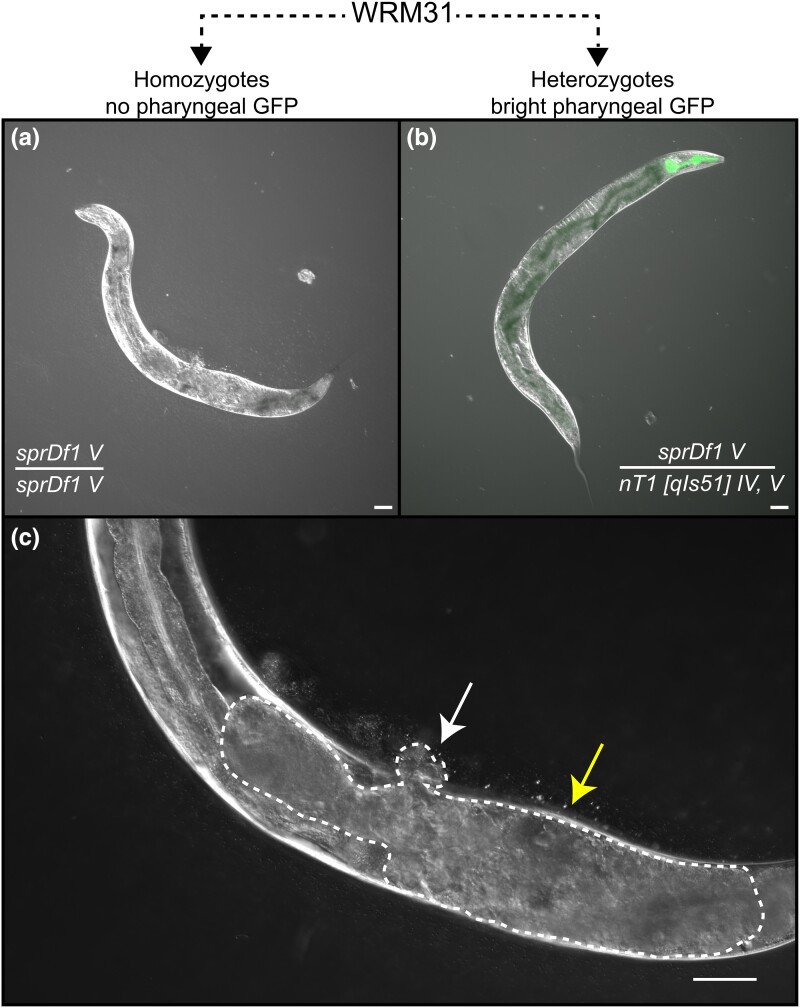
The *sprDf1* microdeletion induces a complex pleiotropic phenotype. The WRM31 strain is a heterozygous animal that contains a microdeletion allele (*sprDf1*) on chromosome V and an *nT1[qIs51]* balancer chromosome that suppresses crossover repair of the deficiency. Only 2 viable genotypes are produced by self-fertilization of this strain: GFP− homozygotes (*sprDf1*/*sprDf1* V) and GFP+ heterozygotes (*sprDf1*/*nT1[qIs51]*). a and b) Adult GFP− *sprDf1* homozygous worms are shorter and thicker (see also Supplementary Fig. 3) than their GFP+ heterozygous siblings and form a large uterine tumor c) that causes a protruding vulva (white arrow) and frequently leads to body wall distention (arrow to the right of the image). The phenotype is easy to follow using standard light microscopy. The scale bar in panels a and b represents a distance of 50 microns, and in panel c it represents 20 microns.

While screening F2 progeny during the process of making a CRISPR knock-in mutation in *C. elegans*, we identified animals with an unusual uterine tumor phenotype (Fig. 1). We mapped the causative mutation (*sprDf1*) to a ∼0.25 Mb microdeletion allele on the left arm of chromosome V that removes 32 adjacent protein-coding genes. Remarkably, homozygous animals survive to adulthood, but are small, sterile, and form large uterine tumors derived from polynucleated misshapen embryos. The animals are uncoordinated and most of them die by bursting within 8 days posthatching. While many deficiency alleles have been genetically mapped, most are embryonic lethal as homozygotes and have primarily been used for mapping and assessing genetic nulls (Sigurdson *et al.* 1984; Rosenbluth *et al.* 1990). Of the 212 multilocus deletion alleles described in Wormbase whose precise genomic coordinates are known, only ten are larger than 10KB, and none are over 50KB (Dubaj Price and Hurd 2019). This 255KB *sprDf1* microdeletion is 5 times larger than the next biggest physically mapped multilocus deletion allele in the worm. As such, it provides a unique opportunity to assess microdeletion-like genetic interactions in a simple model organism. Here, we present the initial characterization of this microdeletion allele as a potential model for the complex genetic interactions induced by similar alleles in humans.

## Materials and methods

### Strains and nematode culture

All strains used in this study were maintained by growing the animals at room temperature on *E. coli*OP50 seeded NGM plates under standard conditions (Stiernagle 2006). The nematode strains used in this study are listed in Table 1. WRM31 was generated by injecting N2 worms with an RNP-mixture containing recombinant *Streptococcus pyogenes* Cas9, a guide RNA targeting *mex-5* (protospacer: UGGAAUCAAACCAUGUGAUA), a second guide RNA targeting *dpy-10* (protospacer: GCUACCAUAGGCACCACGAG), tracrRNA (IDT, Coralville, IA), and a PCR product derived from amplifying DNA encoding the CX10 mutant of mex-5 (Tavella *et al.* 2020) synthesized as a gBlock (IDT, Coralville, IA, Supplementary Table 3) and topo-cloned into a vector using the TA Topocloning kit (Thermo-Fisher, Catalog #451641, Waltham, MA). Primer sequences and gBlocks used in this study are listed in Supplementary Table 3.

**Table 1.** *C. elegans* strains were used in this study.

Strain ID	Genotype
N2	Wild Type (Bristol natural isolate)
VC362	unc-5(e53) (IV)/nT1 [qIs51] (IV;V); dpy-11(e224) (V)/nT1 [qIs51] (V;IV)
CB4856	Wild Type (Hawaiian natural isolate)
WRM31	sprDf1 (V)/nT1 [qIs51] (IV;V)
WRM32	+ (V)/nT1 [qIs51] (IV;V)

**Fig. 2.**
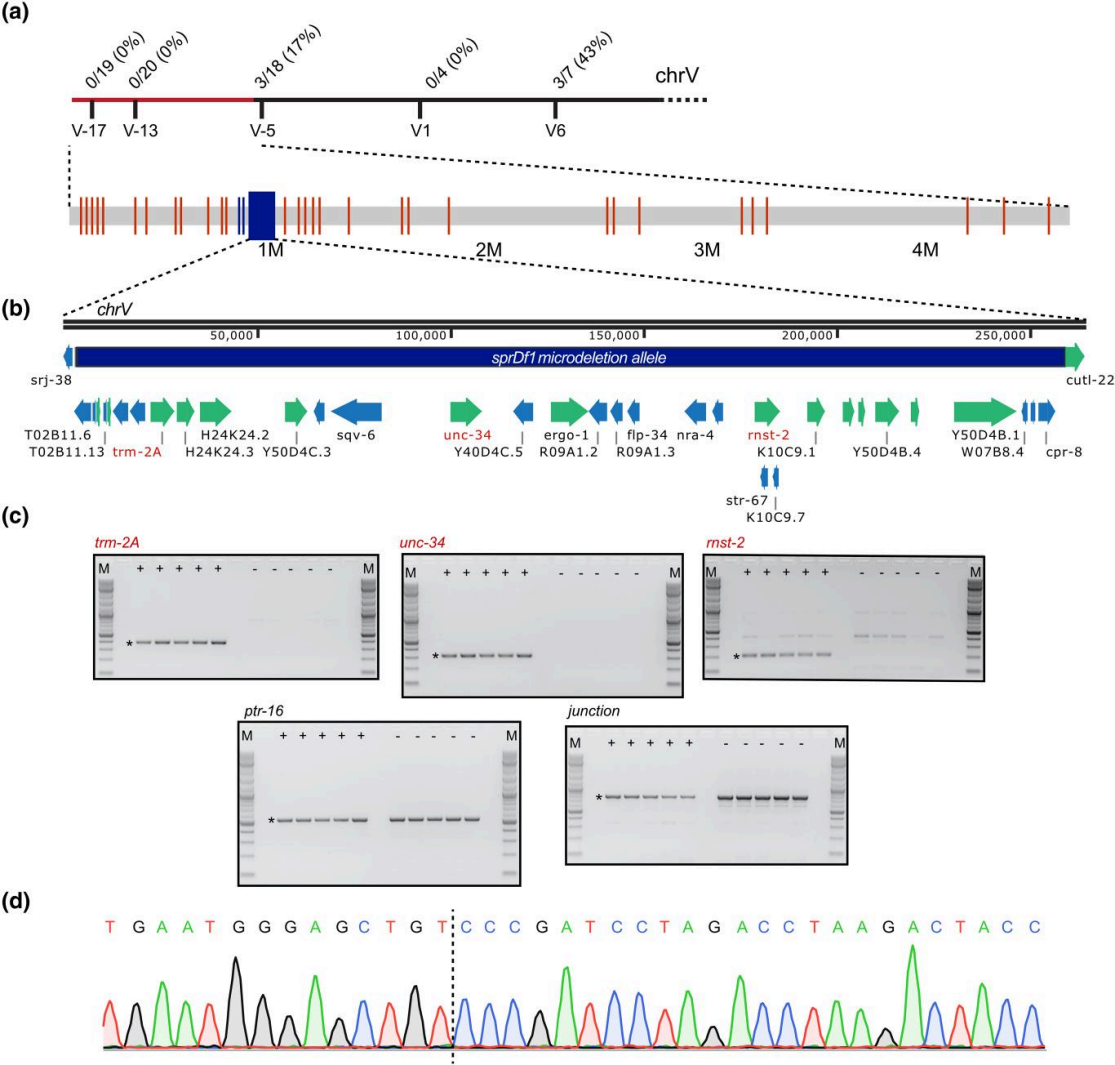
Characterization of the *sprDf1* microdeletion allele. a) Recombination analysis following a cross of WRM31 with the Hawaiin strain (CB4856). The number of recombination events at each SNP is represented by the numerator, while the denominator represents the number of phenotypic cross progeny assessed at each SNP. The mutation lies to the left of SNP snp_Y69A9L (V-5) on chromosome V. This region is shown below with vertical lines indicating the position of WRM31-specific SNPs and Indels detected by whole genome sequencing, and rectangles indicating WRM31 specific SV. b) The *sprDf1* microdeletion allele. The identities and positions of the protein coding genes eliminated in the microdeletion are shown below the map. c) Single worm PCR of multiple GFP+ and GFP− siblings (labeled) to evaluate the presence or absence of 3 genes predicted to be eliminated by the microdeletion (*trm-2A, unc-34,* and *rnst-2*), one gene that lies just outside of the microdeletion allele (*ptr-16*), and with primers that flank the breakpoint predicted in whole genome sequencing of WRM31. In all cases, the expected amplicon product is marked with an asterisk. d) Sequencing trace revealing the precise junction of the microdeletion allele (dashed line).

Candidate mutants were balanced by crossing fertile siblings of animals bearing phenotypic and sterile worms with VC362 (*unc-5(e53)* (IV)/nT1[qIs51] (IV; V); *dpy-11(e224)* V/nT1[qIs51] (IV; V)). F2 animals were isolated, and those that produced only GFP positive wild type-appearing progeny and GFP minus uterine tumor-bearing progeny were propagated. To outcross the balanced WRM31 strain, N2 males were crossed with VC362 hermaphrodites, pharyngeal GFP positive F1 male progeny were subsequently crossed with WRM31 GFP positive hermaphrodites. The F2 progeny that were GFP positive were isolated and allowed to self-propagate, and the presence of the uterine tumor phenotype scored in pharyngeal GFP-negative animals. The crosses were repeated 2 more times, for a total of a 3× outcrossing of the WRM31 strain. The genotype of WRM31 was confirmed by PCR using the primer sets described in Supplementary Table 3 and whole genome sequencing as described in Fig. 2, using the methods outlined below. We note that the outcrossed WRM31 strain does not harbor a mutation in the *mex-5* or *dpy-10* loci. WRM32, which serves as a balancer control, was prepared by crossing WRM31 GFP-positive hermaphrodites with N2 males, and then backcrossing the GFP positive male progeny with N2 hermaphrodites. The GFP positive hermaphrodites from this second cross were isolated and allowed to propagate. The loss of sprDf1 was confirmed with PCR.

### SNP mapping

Recombination mapping experiments were performed by crossing heterozygous WRM31*sprDf1/nT1[qIs51]* hermaphrodites with CB4856 (Hawaiian) males. Hermaphrodite F1 progeny that were wild type (WT) in appearance and lacked GFP were isolated to individual plates and allowed to self-fertilize. F2 progeny that were confirmed to display the uterine tumor phenotype were isolated and then lysed in lysis buffer (30 mM Tris pH = 8, 8 mM EDTA, 100 mM NaCl, 0.7% NP-40, 0.7% Tween-20 + proteinase K) for use in single worm PCR reactions. Lysates were frozen at −80°C for at least 10 min, then incubated at 65°C for 1 hour and 95°C for 15 min before genotyping PCR using the primer sets described by Jorgensen and colleagues (Davis *et al.* 2005). Each PCR product was digested with the DraI restriction enzyme, then run on a 2% agarose gel to visualize products indicative of recombination or lack thereof. The experiments were repeated across multiple animals to determine the frequency of recombination between the mutant and each marker on the Hawaiian strain.

### Whole genome sequencing

Genomic DNA from strains N2, VC362, and WRM31 were prepared using the Qiagen Gentra Puregene Core Kit A (Germantown, MD) according to the manufacturer's instructions. One 60 mm plate of starved worms was used as source material. The quality and concentration of the genomic DNA prep were confirmed using a Qubit fluorometer (ThermoFisher, Waltham, MA). The recovered genomic DNA was sent to Novogene (UC Davis, CA) who prepared libraries, sequenced the genome to an average depth of greater than 15X, and returned mapped data, SNP and indel calls, structural variants (SV), and putative copy number variants (CNV) relative to the reference genome (*C. elegans* ce11). The entire report from Novogene is available as Supplementary Methods. In brief, sequence reads were discarded if either paired-end read was found to contain adapter sequence contamination, 10% or more ambiguous base calls, or Q-score of less than or equal to 5. In total 99.86% of reads were retained after this filtration step. Clean reads were mapped to the C. elegans ce11 reference genome using Burroughs-Wheeler Aligner (BWA) software (Li and Durbin 2009). Duplicates were removed using SAMTOOLS (Li *et al.* 2009). In total, 99.7% of the genome was sequenced to at least 4× coverage for all 3 strains. Single nucleotide polymorphisms and indels were identified using GATK using thresholds given in Supplementary Methods (DePristo *et al.* 2011). SV were identified using BreakDancer (Chen *et al.* 2009). Putative variants with less than 2 supporting paired-end reads were discarded. CNV were also identified using CNVator as described in Supplementary Methods (Abyzov *et al.* 2011).

Single nucleotide polymorphisms, insertions and deletions, copy number variations, and SV were compared between all 3 strains. We refined this list to identify candidate mutations that are (1) covered by the *nT1[qIs51]* balancer, (2) located on chromosome V to the left of snp_Y69A9L, and (3) unique to WRM31. The list can be found in Supplementary Table 2. The presence or absence of these mutations was confirmed by PCR comparing GFP+ to GFP− siblings. Primer sets used to evaluate candidate mutations are listed in Supplementary Table 3. The identity of the breakpoint in the microdeletion was confirmed by Sanger sequencing of the PCR products using primers that flank the breakpoint.

### RNA interference

Knockdown by RNAi was performed by soaking N2 animals in double-stranded RNA corresponding to the cDNA sequence of the *srj-38* gene. We find that soaking animals with dsRNA yields more penetrant phenotypes than RNAi by feeding (Conte and Mello 2003). A synthetic cDNA template which consisted of the srj-38 cDNA harboring T7 promoters was prepared as a gBlock HiFi gene fragment by IDT (Coralville, IA), and then amplified with primers (Supplementary Table 3). The resultant PCR product was used as a template for in vitro transcription (IVT) to produce dsRNA with the Ambion MEGAscript T7 IVT kit (ThermoFisher Scientific cat #: AM1333) following the manufacturer’s protocol. The dsRNA was extracted with phenol-chloroform and then precipitated with isopropanol. RNAi by soaking was performed as previously described (Conte and Mello 2003). Briefly, arrested L1 animals were treated with 4–8 mg of purified dsRNA in soaking buffer at 20°C for 24 hours, then plated onto NGM plates seeded with *E. coli*OP50 and placed in the incubator. Once the animals reached adulthood, the presence or absence of a uterine tumor was scored by inspection using a stereo-dissecting microscope. The presence or absence of polynucleated embryos was also scored by inspection using a 40× DIC objective on a Zeiss AxioObserver 7 motorized inverted microscope. Additionally, brood size assays were performed by separating out previously soaked L4 animals 3 days after plating and counting the total number of embryos and hatchlings each animal produced.

### Brood size, hatch rate, and bursting assays

WRM31 and WRM32 animals were maintained by picking pharyngeal GFP+ heterozygotes under a fluorescence dissecting microscope to limit recovery of animals that lost the balancer chromosome. WRM31 animals were synchronized by dissolving the worms in 20% bleach solution (3 mL concentrated Clorox bleach, 3.75 mL 1 M filtered sodium hydroxide, 8.25 mL filtered MilliQ H2O). Embryos recovered from gravid adults were harvested by centrifugation, washed extensively with M9 salt solution (22 mM KH_2_PO_4_, 42 mM Na_2_HPO_4_, 86 mM NaCl, and 1 mM MgSO_4_), then left in M9 to hatch overnight. L1 larvae were plated on 60 mm OP50-seeded NGM agar plates the following day. Forty synchronized WRM31 GFP+ and GFP− animals were isolated into OP50 seeded NGM agar plates at the L3/L4 stage. The animals were transferred to fresh plates 2 days after singling and transferred to a new plate daily to prevent overcrowding. Prior to transfer, the animals were recorded as alive, burst, or dead. The number of progeny was recorded after transferring worms. The following day, the number of embryos that hatched was recorded. The brood size, hatch rate, fraction burst, and fraction surviving were counted for 8 days. At least 3 biological replicates were used per strain/condition. All data are available in Supplementary Data Set 1.

### In utero imaging of embryo morphology

WRM31 and WRM32 animals were synchronized as described above. On day 4, a subset of animals from each category were mounted in 1 mM levamisole (Sigma-Aldrich, BP212) and imaged using DIC optics on a Zeiss AxioObserver 7 with a 40× objective. Multiple images were collected at different focal planes to capture the dimensions of the embryos. This process was repeated 2 days later with worms from the same synchronization. The presence of polynucleated embryos/cytokinesis failure was determined by looking at the number of nuclei per cell per embryo in the uterus. If embryonic cells contained more than 3 nuclei, they were scored as polynucleated. The fraction of polynucleated embryos present was calculated by dividing the total number of polynucleated embryos by the total number of embryos present. The lengths of the embryos were measured by using the line tool in FIJI to draw a line between the vertices and measuring the major axis. The width of the embryos was determined the same way for the minor axis. The length/width ratio was calculated by dividing the 2 measurements. Older worms were not imaged due to the increased chance of bursting before or during the imaging process.

### Squashed vulva imaging

WRM31 and WRM32 animals were synchronized as described above. Two days after plating the arrested L1 larvae on OP50 seeded NGM agar plates, animals at the L4 stage were isolated, assessed for the presence or absence of pharyngeal GFP, then mounted in 1 mM levamisole and imaged with DIC optics on a Zeiss AxioObserver microscope using a 20× objective. The width of the vulval lumen at the midpoint of the distance between the cuticle and the uterine seam cell (UTSE) valve was measured with the line tool in FIJI as described above.

### Worm velocity and path length assays

WRM31 and WRM32 animals were synchronized via bleaching as described above. Two days after plating L1 larvae (3 days posthatching), worms were separated based on presence of GFP in the pharynx. On day 5 and day 6 posthatching, worms were picked onto unseeded plates and filmed using a a Zeiss Discovery V20 stereo microscope fitted with a Diagnostic Instruments 18.2 Color Mosaic camera. The zoom was 15x. The worms were stimulated with a pick before the images being taken. Images were collected with SPOT software. The movies consisted of 100 frames, taken 0.25 seconds apart, resulting in a 25 second long 4 frame per second movie. The movies were analyzed using the FIJI plug-in wrMTrck (Schindelin *et al.* 2012; Nussbaum-Krammer *et al.* 2015). Before using wrMTrck, the backgrounds were removed using the FIJI tools Z-project and Image Calculator functions, and the image thresholds were calculated with the Otsu algorithm. The wrMTrck outputs (length, average speed) were converted from pixels to microns by using a stage micrometer to determine the number of pixels per micron at 15× (0.2 pixels per micron), and then using the conversion on the outputs.

### Worm size measurements

Synchronized L1 WRM31 animals were plated on OP50-seeded NGM agar plates. Animals were imaged on day 4, 5, 6, and 8 posthatching in 1 mM levamisole using a 5× DIC objective on a Zeiss Axio-Observer microscope. The presence or absence of pharyngeal GFP was determined at the same time by imaging GFP fluorescence. The length of each worm (in microns) was determined using the segmented line tool in ImageJ to draw a line from the tip to the tail through the center line of each animal. The width of each animal was measured using the line tool in FIJI both at the vulva and at the lower bulb of the pharynx. A distention index was calculated by dividing the width of the vulva by the width of the pharynx to normalize for overall size of the animal.

### Statistical analyses

A 1-way ANOVA with Bonferroni correction for multiple hypothesis testing was used to assess statistical significance between genotypes and between time periods for all assays performed, including brood size and hatch rate measurements, polynucleated embryo imaging, vulval lumen measurements, embryo morphology, length, and distension assays. In all cases, data sets plotted on the same axes were compared to all other data sets in the same graph in the ANOVA. For bursting and survival assays, Kaplan Meier logrank *P*-values were calculated for each genotype, and *P*-values were adjusted using the Bonferroni method for multiple hypothesis testing to compare all 4 groups. StatPlus software (AnaylSoft, Inc, Brandon, FL) was used to perform all of the statistical analyses. The output from the ANOVA and Kaplan Meier analyses is included in Supplementary Data Set 1.

## Results

### Recovery of a homozygous viable microdeletion on chromosome v

The original goal of our work was to make a knock-in mutation of the *mex-5* locus using CRISPR with homologous recombination (CRISPR-HR). We sought to mutate the endogenous *mex-5* gene to produce a variant that we previously characterized that increases the thermodynamic stability of the protein fold as measured by NMR (Tavella *et al.* 2020). While analyzing the progeny of CRISPR-injected animals, we identified sterile individuals expressing an unusual uterine tumor phenotype characterized by swelling of the body wall (Fig. 1a). To simplify propagation, we crossed fertile siblings of the phenotypic mutants with VC362, a strain that harbors the nT1 [qIs51] IV; V reciprocal translocation chromosome that acts as an effective balancer for the *mex-5* locus. Homozygous nT1 [qIs51] IV; V animals die as larvae due to the presence of an uncharacterized lethal mutation (Edgley *et al.* 2006). Heterozygous animals grow to adulthood, are fertile, and are easily identified under a fluorescence microscope through a bright pharyngeal GFP transgene integrated on the balancer chromosome (Edgley *et al.* 2006) (Fig. 1b). Animals homozygous for the mutation lack the balancer chromosome and thus pharyngeal GFP, simplifying identification of homozygous mutant self-progeny.

In the process of outcrossing this strain through wild-type N2 worms, we discovered that the unusual uterine tumor phenotype persists in animals that no longer harbor the mutant *mex-5* allele, suggesting the phenotype derives from a co-occurring mutation also balanced by nT1[qIs51]. Being intrigued by the phenotype, we decided to map the causative locus by crossing the mutant, made in the N2 background, with the divergent Hawaiian strain (CB4856) that contains numerous SNPs that facilitate recombination mapping (Davis *et al.* 2005). This analysis revealed the mutation is located to the left of snp_Y61A9LA/WBVar00208343 (recombination frequency 16.7%) on Chromosome V (Fig. 2a).

To further identify candidate mutations, we next performed whole genome sequencing of DNA prepped from mixed-stage animals of the parental N2 strain, VC362 (the source of the nT1[qIs51] balancer chromosome), and WRM31, which harbors the balanced candidate mutation (Fig. 1). This analysis identified 39 SNPs or short indels unique to WRM31 that map to the left arm of chromosome V (Fig. 2b, Supplementary Table 1). Thirty-seven have an allele frequency of ∼0.5, as expected for a heterozygous allele. Of these, 15 are intergenic, 10 are within introns, 9 are upstream or downstream of a known or predicted gene, 2 are within annotated UTRs, and 1 is in the exon of a noncoding RNA gene. None are found within a protein-coding exon.

Analysis of potential structural variations identified 3 additional candidates unique to WRM31 on the left arm of chromosome V that perturb one or more open reading frames. The candidates are a 633 base pair deletion within K09C6.9, a 1.02 kilobase pair deletion within *srj-38*, and a 255 kilobase pair microdeletion predicted to disrupt 32 protein-coding genes. To validate the presence or absence of the monogenic K09C6.9 and *srj-38* deletions in WRM31, we designed primer sets to detect the predicted deletions and performed single worm PCR comparing GFP− phenotypic animals to GFP+ nonphenotypic siblings. The results confirmed the presence of the deleted region in *srj-38* and K09C6.9 (Supplementary Fig. 1a). However, phenotypic animals also harbored full-length K09C6.9, and a shorter band of unknown origin. The presence of an intact copy of the gene in phenotypic animals suggests that the phenotype is not due to loss-of-function of this gene. The deletion removes the promoter and part of exon 1, including the translation start site. K09C6.9 has no known function, and no phenotypes have been reported in 37 mapped alleles (Thompson *et al.* 2013). Consistent with this hypothesis, the entire gene, along with several adjacent genes (including *srj-38*), is deleted in a natural isolate of C. elegans from Adelaide, Australia (AB1) (Thompson *et al.* 2013).

SRJ-38 is a 7 transmembrane domain G-protein coupled receptor that is only expressed during the alternative dauer stage larval (Supplementary Fig. 1b) cycle. This protein has no known function. The deletion allele we recovered removes most of exon 1, all of exon 2, and most of exon 3, disrupting 6 out of the 7 predicted transmembrane domains. As such, we predict this allele likely eliminates the function of SRJ-38. There are 24 mapped alleles of this gene, and 46 naturally occurring variants, none of which have been shown to harbor a phenotype. In addition, no phenotypes have been reported upon knockdown in previous genome-wide RNAi screens. Consistent with prior findings, we did not observe reads mapping to *srj-38* mRNA in mixed population wild-type N2 worms in 6 previously published RNAseq data sets collected by our lab (Albarqi and Ryder 2021), nor could we amplify this transcript by RT-PCR assays from the mutant or wild-type controls. Consistent with previous findings, we observe no tumor phenotype, no reduction in brood size or hatch rate, and no embryonic phenotypes upon soaking N2 worms with dsRNA targeting *srj-38* mRNA (Supplementary Fig. 1). This suggests the unusual uterine tumor phenotype is not caused by the deletion allele within the *srj-38* locus.

To test whether the 255KB microdeletion allele is present in mutant animals as predicted by the whole genome sequencing data, we designed primer sets to detect 3 genes that lie within the microdeletion (*rnst-2*, *unc-34*, and *trm-2A*), and another that lies just outside of the microdeletion breakpoint (*ptr-16*) as a control. PCR of single worm lysates made from heterozygous GFP+ worms robustly detect all 4 amplicons, while PCR of lysates made from phenotypic GFP− siblings detect *ptr-16* (Fig. 2c), but not *rnst-2*, *unc-34,* or *trm-2*, consistent with the presence of a large deletion. To further confirm the deletion allele, we designed primers flanking the predicted break-point junction. These primers only amplify the deletion allele yielding a predicted product of 768 base pairs. Consistent with the presence of a large microdeletion allele, we observed an ∼800 base pair band in both GFP+ heterozygotes and GFP− homozygotes (Fig. 2c). We cloned and sequenced this PCR product, which identified the precise microdeletion junction. The left side break-point lies within exon 6 of the T02B11.6 locus, and the right side lies within an intergenic region between *cpr-8* and *cutl-22* (Fig. 2d). In total, 255,603 base pairs are lost in this deletion, eliminating 32 characterized or predicted protein-coding genes, including genes with known functions in reproduction (Supplementary Table 2). We suggest that the microdeletion allele is likely to cause the phenotypes observed.

### The microdeletion induces a uterine tumor phenotype

**Fig. 3.**
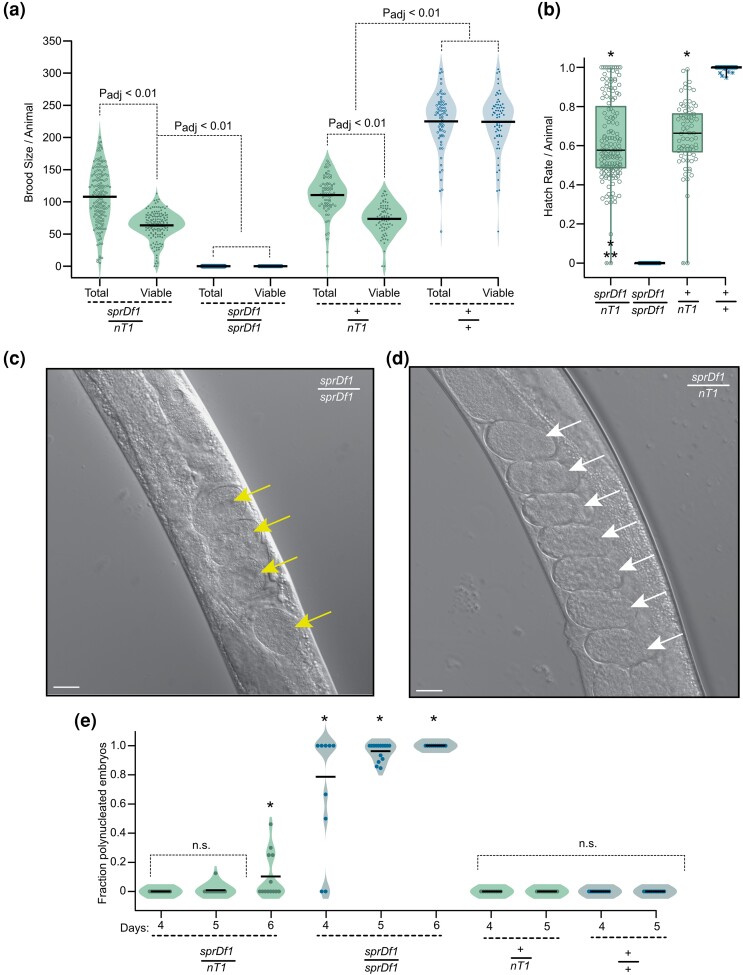
The *sprDf1* allele induces a maternal effect lethal phenotype with polynucleated misshapen embryos. a) The total brood size was determined by counting the number of embryos and animals deposited on a plate by a single hermaphrodite adult throughout its fertile life span. The total viable brood was determined by counting the number of embryos that hatched into animals that survived to at least the L2 stage. The data are represented as violin plots. The genotype is given below the graph. Open circles correspond to the total brood produced by a given animal. Closed circles correspond to the viable brood. The bar denotes the mean. Statistical significance was assessed using a 1-way ANOVA comparing all samples, total and viable, with Bonferroni correction for multiple hypothesis testing. The abbreviation *nT1* is used in place of *nT1[qIs51]* throughout the figure. b) The hatch rate was calculated by dividing the number of viable animals by the total brood. The box represents the quartiles 1–3, and the whiskers denote the full range of the data. Open circles represent the hatch rate per adult hermaphrodite. Stars represent far outliers, and circles with an X represent near outliers as calculated by the Tukey method. The black bar represents the median. A single asterisk denotes P_adj_ < 8.3e-3 comparing all data sets to +/+, and a double asterisk represents a significant decrease with P_adj_ < 8.3e-3 comparing all data sets to *sprDf1/nT1[qIs51]* in a 1 way ANOVA with Bonferroni correction for multiple hypothesis testing. c) Representative micrographs of young adult *sprDf1/sprDf1* homozygotes and *sprDf1/nT1[qIs51]* heterozygotes collected 4 days post hatching. The yellow arrows indicate polynucleated embryos, while the white arrows represent embryos that appear normal. The scale bars represent a distance of 20 microns. d) The fraction of polynucleated embryos retained in utero per animal was determined at day 4, 5, or 6 post hatching. The mean is denoted by a horizontal black bar. The *P*-values were calculated by 1-way ANOVA with post hoc Bonferroni correction for multiple hypothesis testing across all genotypes and all days measured. A single asterisk represents a statistically significant difference with adjusted *P*-value < 1.1e-3 with reference to the day 4 +/+ control.

As the mutation most likely to cause the phenotype is a large microdeletion, we have designated the allele *sprDf1* according to convention (WRM31: *sprDf1 (V)/nT1[qIs51] (IV, V),* Table 1. Animals homozygous for *sprDf1* display a wide array of phenotypes. Most notably, *sprDf1/sprDf1* animals form large, disorganized tumors within the uterus after the last larval molt (Fig. 1). These apparent tumors grow to a large size causing distension of the body wall. To quantitatively assess these phenotypes, we measured the total brood (number of embryos laid on the plate), the number of viable progeny, and the hatch rate for each animal in both genotypes (Fig. 3a). Of the 180 *sprDf1/sprDf1* animals scored across 3 biological replicates, all were sterile, lacking the ability to deposit eggs on the plate. No viable hatchlings were retained in the uterus, though in all cases the uterus was filled with apparent mishappen embryos that grew to large size. In contrast, 153 out of 155 GFP+ *sprDf1/nT1[qIs51]* animals scored were fertile and produced viable progeny. The worms produced an average of 108 ± 43 embryos and 63 ± 22 viable progeny per isolate, yielding an average hatch rate of 58 ± 21%.

To assess the contribution of the balancer chromosome, we prepared a control strain harboring the balancer chromosome but not the microdeletion allele (WRM32: *+ (V)/nT1[qIs51] (IV, V).* The brood size, number of viable progeny produced, and the hatch rate of *sprDf1/nT1 [qIs51]* animals are indistinguishable from that of *+/nT1[qIs51]* (Fig. 3, *n* = 75, P_adj_ = 1 (brood), P_adj_ = 0.48 (viable), P_adj_ = 0.66 (hatch rate)). By contrast, +/+ animals produced significantly more progeny, all of which were viable (Fig. 3, *n* = 67, P_adj_ < 0.00179 brood, viability, and hatch rate). The results suggest that the sterility phenotype observed in *sprDf1/sprDf1* animals is recessive, while the reduction of brood observed in *sprDf1/nT1[qIs51]* and *+/nT1[qIs51]* compared to +/+ is likely due to the balancer chromosome and not the microdeletion allele. The full distributions of total brood, viable brood, and hatch rate for all 4 genotypes are presented in Fig. 3, a and b.

### Homozygous microdeletion mutants form misshapen polynucleated embryos

To better characterize the origin of the uterine tumor, we synchronized animals of all 4 genotypes and imaged the uterus from day 4 through day 6 posthatching. At day 4 post hatching, 80% of the *sprDf1/sprDf1* animals retained polynucleated embryos in their uterus (8/10 animals imaged, Fig. 3, c and e) while none of the embryos observed in GFP+ *sprDf1/nT1[qIs51]* animals appeared to be polynucleated (Fig. 3, d and e, *n* = 0/14). On day 5, the fraction of *sprDf1/sprDf1* animals harboring polynucleated embryos increased to 100% (*n* = 15/15), while 6.25% (*n* = 1/16) of heterozygous siblings retained polynucleated embryos (Fig. 3e). By day 6, all homozygous mutants (*n* = 11/11) retained polynucleated embryos and the fraction of heterozygous animals retaining polynucleated embryos grew to 38% (*n* = 5/13). By contrast, polynucleated embryos were not observed in age-matched *+/nT1[qIs51]* balancer control animals or *+/+* homozygous siblings (*n* = 24, *n* = 25, respectively), with the caveat that retained embryos could not be visualized on day 6 as these animals had exhausted their brood. The presence of polynucleated embryos in *sprDf1/nT1[qIs51]* heterozygotes but not in +*/nT1[qIs51]* balancer control animals suggests that the polynucleated embryo phenotype is not caused by the balancer.

The embryos produced by GFP− *sprDf1/sprDf1* animals were unusually round compared to GFP− *sprDf1/nT1[qIs51]* heterozygous embryos, with an average length-to-width ratio of 1.4 ± 0.2 compared to 1.7 ± 0.3 on day 4 (P_adj_ < 8.3e-3, Supplementary Fig. 2). By day 6, the GFP− *sprDf1/sprDf1* mutants and their GFP+ *sprDf1/nT1[qIs51]* heterozygous siblings both contained unusually round embryos, consistent with both genotypes displaying embryonic defects at this age (GFP− = 1.3 ± 0.2, GFP+ = 1.5 ± 0.3, P_adj_ GFP+ day 4 vs day 6 < 8.3e-3, Supplementary Fig. 2).

### Mutants die by bursting at a young age

**Fig. 4.**
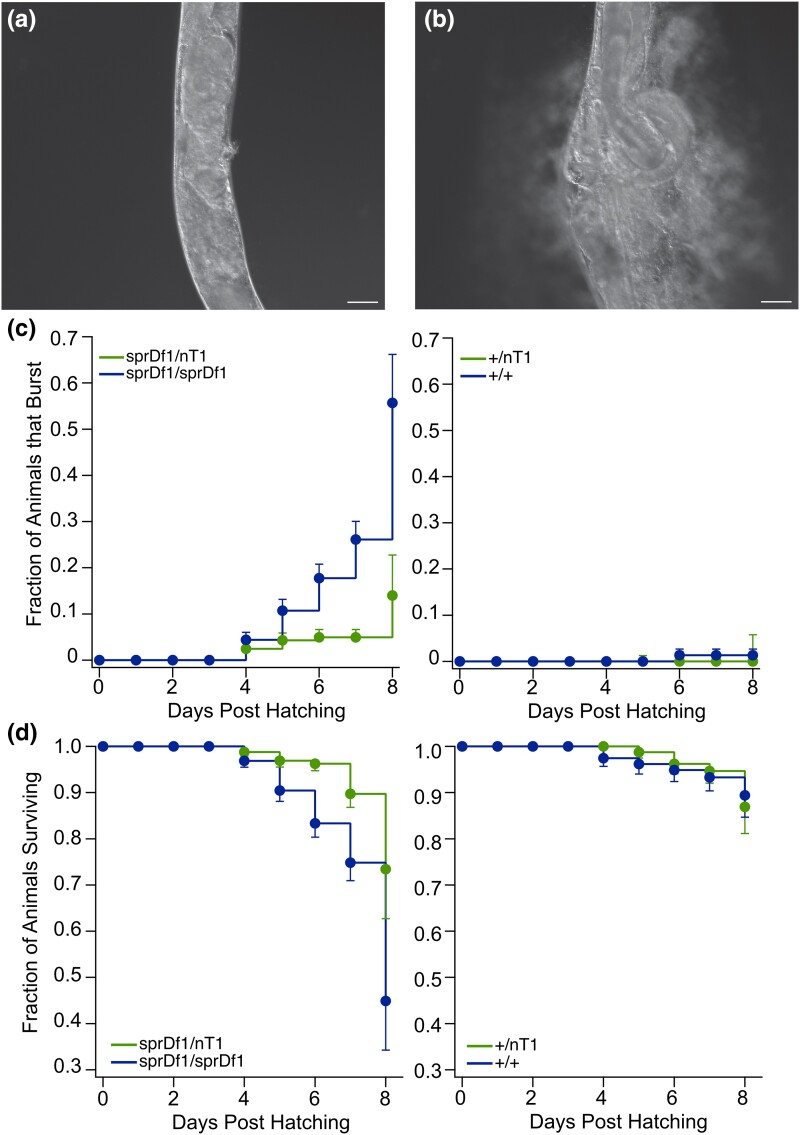
Microdeletion *sprDf1* mutants are prone to death by bursting. DIC micrographs of a GFP− *sprDf1/sprDf1* homozygous worm observed while bursting are shown. The full movie is available as Supplementary Movie 1. a) This frame corresponds to time zero, the beginning of the movie. b) This frame corresponds to the same animal, 4 minutes and 57 seconds later. The scale bars in both panels represent a distance of 20 microns. c) The fraction of animals that died by bursting plotted as a function of days posthatching is shown with GFP+ heterozygotes and GFP− homozygous siblings. The left panel compares *sprDf1/spDf1* homozygotes to *sprDf1/nT1[qIs51]* heterozygotes, while the right panel shows both *+/+* and *+/nT1[qIs51]* genotypes. The data shown represent the sum of multiple synchronizations, with N ranging from 28 to 166 animals per genotype per day. The abbreviation *nT1* is used in place of *nT1[qIs51]* throughout the figure. The error bars represent the standard error. Statistical significance was determined by Kaplan Meier estimation using the logrank method with StatPlus software and Bonferroni correction for multiple hypothesis testing. d) The fraction of animals surviving is represented as in panel c for all 4 genotypes.

To determine whether the microdeletion mutation reduces the life span of the worm, we compared homozygous *sprDf1/sprDf1* animals to *sprDf1/nT1[qIs51]* heterozygous siblings. Eight days after hatching, 45% of *sprDf1/sprDf1* animals and 73% of *sprDf1/nTq[qIs51]* siblings survived (P_adj_ = 0.0003, Fig. 4). Strikingly, in both genotypes, a significant number of animals died by bursting (Fig. 4, a–c, Supplementary Movie 1). By day 8, 55% of *sprDf1/sprDf1* animals died by bursting, while 14% of *sprDf1/nT1[qIs51]* animals died by bursting (P_adj_ = 2.2e-6, Fig. 4c). The cause of death for the remainder of the animals was not determined. Only 1 animal of the 160 animals scored between control genotypes died by bursting, and *+/nT1[qIs51]* animals survived longer than *sprDf1/nT1[qIs51]* heterozygotes (P_adj_ = 0.017) but did not show a statistically significant reduction in lifespan compared to +/+ siblings (P_adj_ = 0.30). As such, the balancer chromosome does not appear to contribute to the reduced lifespan or bursting phenotypes.

### The microdeletion mutation causes a squashed vulva phenotype

**Fig. 5.**
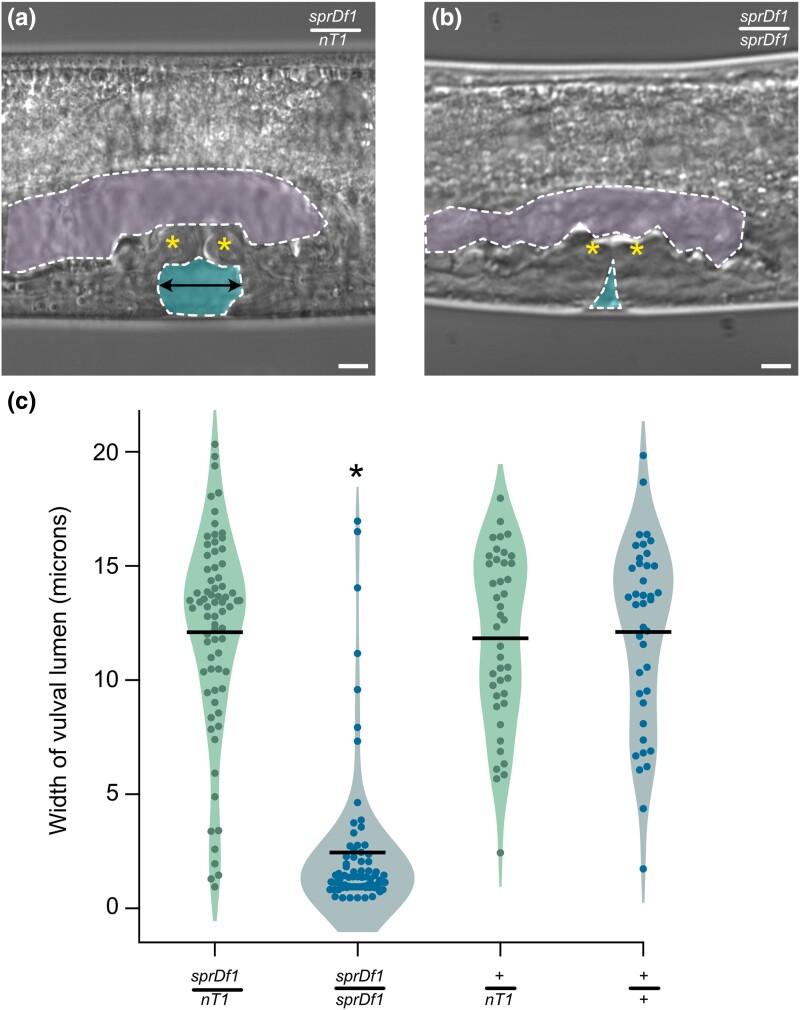
*sprDf1* homozygotes have a squashed vulva and are shorter and wider than their heterozygous siblings. a) DIC micrograph of an L4 *sprDf1/nT1[qIs51]* heterozygote showing the developing vulva. The vulval lumen is indicated by the lower cyan shape, and the uterus is indicated by the upper purple shape. The UTSE valve which separates the uterus from the vulva is indicated by asterisks. The arrow denotes where the measurement is made to define the width of the vulval lumen b) Equivalently aged L4 homozygous *sprDf1/sprDf1* worms display a dramatic reduction in the width of the vulval lumen (a *sqv* or *squashed vulva* phenotype), and the UTSE valve does not form properly (asterisks). In panels a and b, the scale bar represents 5 microns. c) The distribution of luminal widths of the vulva is shown for homozygous animals vs heterozygous siblings for *sprDf1/sprDf1*, *sprDf1/nT1[qIs51]*, +/+, and *+/nT1[qIs51]* genotypes. Statistical significance was calculated from a 1-way ANOVA with Bonferonni correction for multiple hypothesis testing. An asterisk indicates an adjusted *P*-value of <8.3e-. Each point represents the luminal of from a single animal, and bars represent the mean. The abbreviation *nT1* is used in place of *nT1[qIs51]* throughout the figure.

We were intrigued by the bursting phenotype and wondered if vulva morphogenesis defects contribute. Notably, the microdeletion eliminates the *sqv-6* gene, which had previously been identified in a forward genetic screen for the squashed vulva phenotype. Previously characterized *sqv-6(n2845)* mutants display a striking decrease in the vulval lumen volume during the larval L4 stage and defects in the formation of the uterine seam cell (UTSE) valve between the uterus and the vulva (Herman *et al.* 1999). To assess whether the microdeletion induces a similar phenotype, we imaged the vulva of L4 homozygous and heterozygous animal and measured the width of the vulva lumen at the midpoint between the UTSE valve and the cuticle. Consistent with loss of *sqv-6*, *sprDf1/sprDf1* homozygotes had a mean luminal width 2.4 ± 3 μM (*n* = 73), while the luminal width of *sprDf1/nT1[qIs51]* siblings was 5 times larger on average (12.1 ± 4 μm, P_adj_ < 8.3e-3, *n* = 76), Fig. 5, a–c), similar to balancer control (*+/nT1[qIsS5])*, 11.8 ± 4, P_adj_ < 8.3e-3, *n* = 42) and wild type (+/+. 12.1 ± 4, P_adj_ < 8.3e-3, *n* = 40) animals. The results are consistent with a model where recessive loss of *sqv-6* in the *sprDf1* homozygotes is responsible for the squashed vulva allele, with no contribution from the balancer. The distributions are displayed in Fig. 5c.

### Homozygous microdeletion mutants are uncoordinated

**Fig. 6.**
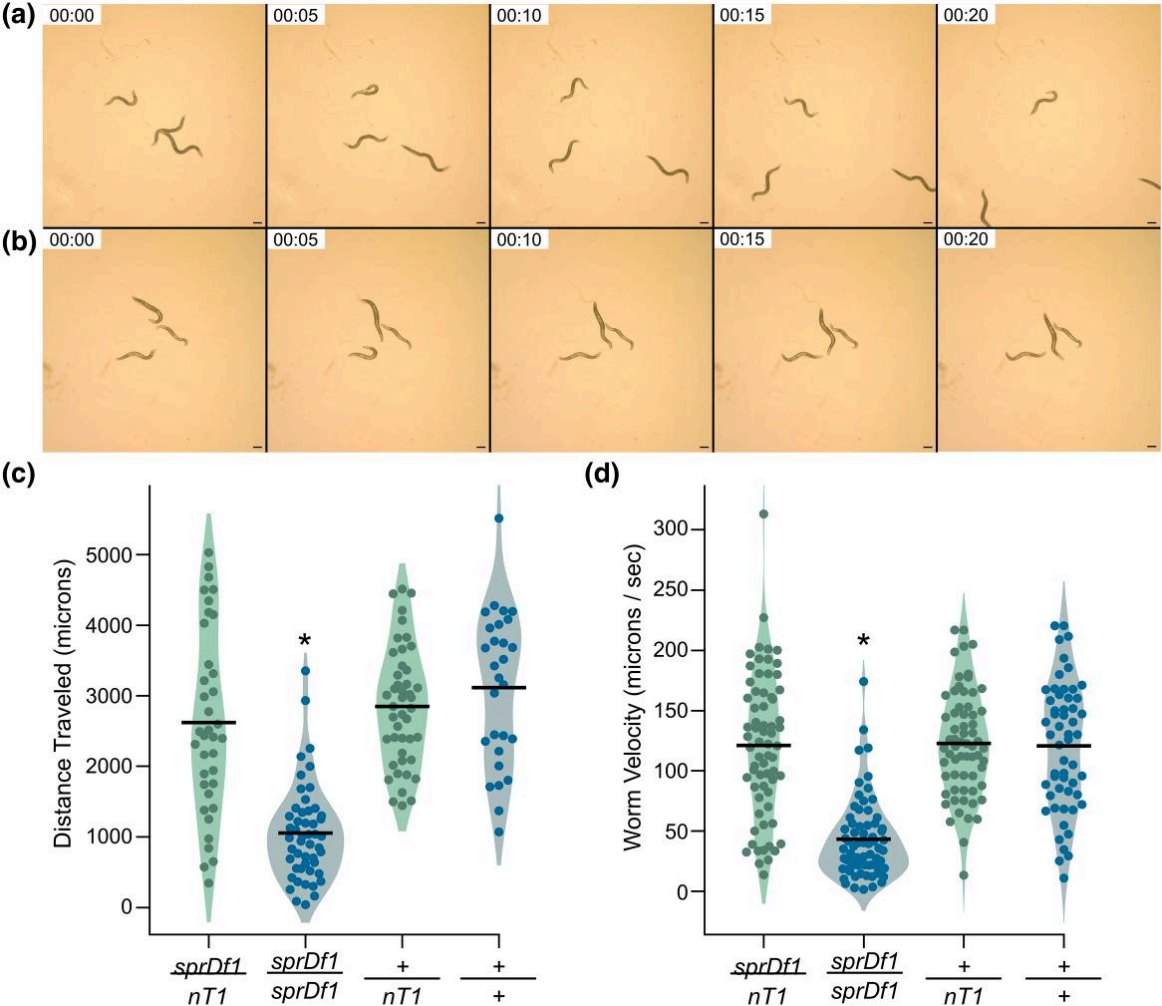
Homozygous microdeletion mutant animals are uncoordinated. a and b). Still frames from movies collected for GFP+ *sprDf1/nT1[qIs51]* heterozygous worms (top row) and GFP− *sprDf1/sprDf1* homozygous worms (bottom row). The full movie can be seen in Supplementary Movie 2. The time stamps indicate time post initiation of filming, and the scale bar represents 200 microns. c and d). The total length of the path traveled (c) and the rate of travel is shown. Each point represents the distance traveled by an individual animal and bars represent the mean. The genotypes and animals’ ages are shown below. Statistical significance between genotypes was calculated with a 1-way ANOVA with Bonferroni post hoc correction for multiple hypothesis testing, where a single asterisk denotes P_adj_ < 8.3e-5. The abbreviation *nT1* is used in place of *nT1[qIs51]* throughout the figure.

Another striking phenotype observed in animals homozygous for the microdeletion is strongly uncoordinated movement. Adult animals become progressively paralyzed as they age. Larval animals and young adults appear to move relatively normally, but older animals have difficulty moving their tails, and do not travel far on a standard NGM-agar plate (Fig. 6, a and b, Supplementary Movie 2). To quantitate these phenotypes, we filmed movement of all 4 genotypes across 25 seconds as described in the methods. Heterozygous *sprDf1/nT1[qIs51]* worms traveled 2.5 times further on average than their homozygous *sprDf1/sprDf1* siblings (Fig. 6, c and d, *sprDf1/nT1[qIs51]* = 2600 ± 1300 μm, *n* = 37; *sprDf1/sprDf1* = 1100 ± 700 μm, *n* = 50, P_adj_ < 8.3e-3). The *sprDf1/nT1[qIs51]* worms also traveled at a higher rate of speed than *sprDf1/sprDf1* homozygous siblings (*sprDf1/nT1[qIs51]* = 120 ± 60 μm/sec, *n* = 64; *sprDf1/sprDf1* = 40 ± 30 μm/sec *n* = 55, P_adj_ < 8.3e-3). There were no significant differences between *+/nT1*, *+/+*, and *sprDf1/nT1[qIs51]* animals in distance traveled or velocity, indicating the uncoordinated phenotype requires 2 copies of the microdeletion allele.

### Homozygous microdeletion mutants are short and become distended with age

Animals homozygous for the microdeletion appeared both shorter and wider than their heterozygous siblings. Between day 3 and day 6 post hatching, homozygous *sprDf1/sprDf1* animals were 10–20% shorter than heterozygous *sprDf1/nT1[qIs51]* siblings (Supplementary Fig. 3a, P_adj_ = 2.18e-12). To quantify the apparent thickening of homozygous animals compared to heterozygous siblings, we divided the vulval width measurement by the width of the lower bulb of the pharynx to define a distension index that could be compared across siblings of both genotypes (Supplementary Fig. 3b). This index revealed that homozygous *sprDf1/sprDf1* animals were 1.2-fold thicker on average than heterozygous *sprDf1/*siblings (P_adj_ = 4.5e-8). The thickening manifests in older animals and is likely caused by the unregulated growth of the embryonic tissue in the uterus.

## Discussion

How simultaneous haploinsufficiency of many adjacent genes leads to strong yet variable phenotypes is not well understood and has not been directly assessed in a simple model organism. We recovered a 255KB multilocus microdeletion (*sprDf1)* in *C. elegans* analagous microdeletion alleles in a variety of human diseases (Watson *et al.* 2014; Sullivan 2019). Mutants that harbor this microdeletion are viable and fertile as heterozygotes, and survive to adulthood, yet are sterile as homozygotes, and display multiple striking phenotypes in both homozygotes and heterozygotes.

Reminiscent of the human microdeletion syndromes, heterozygotes display widely disparate phenotypic penetrance, even among siblings from the same brood. These phenotypes include the formation of polynucleated embryos, shortened lifespan, and death by bursting, which are fully penetrant in homozygotes but only partially penetrant in heterozygotes, suggesting incomplete dominance. We suggest that the nematode strain described in this study provides a useful model to investigate the complex relationships between pathways that are simultaneously disrupted in microdeletion-like alleles. For example, to what extent does of loss-of-heterozygosity contribute to variable phenotypic penetrance? This could be measured directly in this strain because it's possible to produce a large population of heterozygotic siblings where both the magnitude of the phenotype and the genotype could be scored for each individual. In addition, the mutation described in this work enables genetic enhancer and suppressor screens that could identify mutations that modify the penetrance or magnitude of the observed phenotypes. The relative ease of performing forward and reverse genetic screens in this species would facilitate such efforts. Finally, the impact of environmental exposure to mutagens, fluctuating temperature, or changes in diet could be measured using the quantitative assays outlined here. And while *C. elegans* is evolutionarily distant from humans, and there is no clear synteny between this microdeletion and any specific microdeletion syndrome in humans, the robust genetics, rapid gene disruption technology, and simple RNAi screening enable a more thorough characterization than would be feasible in humans or mammalian model organisms.

### Genes perturbed in the sprDf1 mutation

The 255KB *sprDf1* microdeletion removes 32 adjacent protein coding genes, including 16 that have not been studied and 16 more where we have some information about their function from the literature. Of these, genetic screens have identified phenotype-inducing alleles in 6 of them, 4 of which also show phenotypes in RNAi screens. These genes are *rnst-2*, *nra-4*, *ergo-1*, *unc-34*, *sqv-6*, and *fmo-5.* It is unlikely that deletion of a single gene within the microdeletion accounts for all of the phenotypes observed in *sprDf1* homozygotes. Some phenotypes, including squashed vulva and uncoordinated movement, appear to be recessive. Others, including the presence of polynucleated embryos, premature death, and bursting, appear to be incompletely dominant. This strain provides an opportunity to dissect out which phenotypes correspond to loss of which genes, or sets of genes. While the precise genetic basis of each phenotype remains unclear, prior studies suggest some intriguing possibilities, outlined below.

### Genes that potentially contribute to death by bursting

Both *sprDf1/nT1[qIs51]* heterozygous and *sprDf1/sprDf1* homozygous animals are prone to premature death by bursting. We suggest this phenotype is likely caused by loss of a combination of genes. One key feature of the bursting phenotype appears to be unregulated growth of uterine tumors derived from polynucleated embryos that lead to distension of the body wall. The presence of polynucleated embryos in the microdeletion mutant could be attributable to disruption of the *sqv-6* gene. This gene encodes *C. elegans* sole homolog of XYLT1, a polypeptide xylosyltransferase required for proteoglycan synthesis (Hwang *et al.* 2003). The only characterized allele (*n2845*) is a nonsense mutation that results in loss of 42 amino acids on the c-terminus of SQV-6 (Herman *et al.* 1999; Hwang *et al.* 2003). On its own, the *n2845* allele does not cause bursting. The *sqv-6(n2845)* mutation leads to embryonic arrest at the one-cell stage without nuclear division (Hwang *et al.* 2003). However, polynucleated embryos have been observed in published RNAi studies of *sqv-6*, and are also observed in other *sqv* gene mutants involved in proteoglycan synthesis (Wang *et al.* 2005). The unregulated growth of these embryos into a uterine tumor, and subsequent bursting of the animals, was not observed in either the *n2845* mutant or in *sqv-6* RNAi animals. As such, it is likely that disruption of additional genes in the microdeletion further contributes to the observed phenotypes.

Another gene that potentially contributes is *nas-32*. This gene encodes an astacin-like metalloprotease endopeptidase involved in molting. Published studies describing loss-of-function mutations and/or RNAi phenotypes of related family members revealed defects in the molting cycle, suggesting a role for these enzymes in modifying the architecture of the cuticle (Mohrlen *et al.* 2003; Suzuki *et al.* 2004). It could be that the unregulated growth of embryonic tissue in the uterus coupled with defects in the cuticle combine to cause premature death by bursting. Other genes lost in the microdeletion could also contribute to the phenotype, including additional factors potentially involved in proteoglycan carbohydrate remodeling (*clec-203*, Y50D4B.4) and additional proteases related to cathepsin B (*cpr-5* and *cpr-8*). These hypotheses remain to be directly tested.

### Vulva morphogenesis defects

Vulval morphogenesis defects are observed in homozygous *sprDf1/sprDf1* animals but not in heterozygous siblings or balancer control animals. As such, this phenotype appears to be recessive. It is likely that this defect is also caused by loss-of-function of the *sqv-6* gene, which was previously recovered in a screen for the squashed vulva (*sqv*) phenotype. The *sqv-6(n2845)* allele causes a vulval morphogenesis phenotype identical to that shown in Fig. 5b. It is reasonable to assume that uterine retention caused by vulva morphogenesis defects contributes to the bursting phenotype. Indeed, the bursting phenotype is more penetrant in *sprDf1/sprDf1* homozygotes than in heterozygous *sprDf1/nT1[qIs51]* siblings, and we do not observe a statistically significant difference in vulva luminal measurements between *sprDf1/nT1[qIs51]* animals and *+/nT1[qIs51]* control animals. As such, vulval morphogenesis defects may contribute to the bursting phenotypes, but it is not absolutely required. More work is necessary to tease apart the interdependency between polynucleated embryos, bursting, and vulva morphogenesis defects.

### Genes that potentially contribute to shortened lifespan

The *rnst-2* gene encodes a homolog of the ribosomal RNA T2 endonuclease required for autophagy of ribosomes in the lysosome. Loss of *rnst-2* activity leads to partially penetrant maternal-effect embryonic lethality (20–30%), larval arrest (50%), and a shortened lifespan (Liu *et al.* 2018). Animals deficient in *rnst-2* accumulate ribosomal RNA and have larger than normal lysosomes. While *rnst-2* mutants share some features of the *sprDf1*/*sprDf1* and *sprDf1/nT1[qIs51]* animals, including shortened lifespan, there is no evidence that *rnst-2* mutants die by bursting, and the embryonic lethality phenotype of the *sprDf1* homozygotes is much more penetrant (100%) than *rnst-2* null mutants alone. It is possible that loss of *rnst-2* is responsible for premature death not caused by bursting in the microdeletion mutants, but it remains possible that other genes lost in the microdeletion also contribute to lifespan.

The *nra-4* gene encodes a transmembrane domain protein that associates with nicotinic acetylcholine receptors in the endoplasmic reticulum and regulates their assembly in synapses (Almedom *et al.* 2009). It is homologous to nodal modulator (NOMO), an agonist of TGFβ signaling pathways in vertebrate embryos. Hypomorphic alleles of this gene cause a variety defects in *C. elegans*, including mild resistance to aldicarb, levamisole, and nicotine, morphology defects at synapses, as well as a reduced brood size. RNAi of *nra-4* revealed a very low penetrance embryonic lethality phenotype (0.83%) as well. The mild phenotypes reported for this gene suggest that it alone does not account for the strong phenotypes reported above for the *sprDf1* microdeletion, although it is possible that loss of *nra-4* contributes to the reduced brood observed.

The *ergo-1* gene encodes an Argonaute family protein that associates with a subpopulation of 26G endogenous siRNAs that appears to play a role in silencing pseudogenes, recently duplicated genes, and long noncoding RNAs (Vasale *et al.* 2010). Loss of *ergo-1* mutants enhance RNAi phenotypes, but otherwise displays no other physiological phenotype, and as such loss of the *ergo-1* gene is unlikely to contribute to the striking phenotypes of *sprDf1* described above.

### Genes that potentially contribute to the uncoordinated phenotype

The strong uncoordinated movement/tail paralysis phenotype observed in *sprDf1* homozygotes is likely due to loss of the *unc-34* gene. Loss of function mutations in *unc-34* display a severe and fully penetrant uncoordinated phenotype characterized by paralysis, withered tails, and/or dramatic reduction in mobility relative to wild-type animals (Yu *et al.* 2002; Withee *et al.* 2004; Fleming *et al.* 2010). UNC-34 encodes the lone homolog of Enabled/VASP (Vasodilator-stimulated phosphoprotein) in the *C. elegans* genome. Ena/VASP proteins promote the assembly of F-actin filaments in a variety of contexts (Krause *et al.* 2003). In *C. elegans*, loss of *unc-34* disrupts the migration of several neuronal cell types, including CAN, PVQ, HLN, AVM, VD, and DD neurons (Yu *et al.* 2002; Fleming *et al.* 2010). In addition to *unc-34*, the *sprDf1* microdeletion also removes several uncharacterized genes that are expressed or enriched in neurons, including K10C9.7, K10C9.1, Y50D4B.2, and Y50D4B.1. The microdeletion also disrupts *flp-34*, which encodes a neuropeptide involved in olfactory learning processes (Fadda *et al.* 2020). We cannot rule out that the loss of these genes contributes to the uncoordinated phenotype. Nor can we rule out that the uncoordinated movement/paralysis could be secondary to the unregulated growth of uterine tumor tissue, which occupies much of the body cavity.

### Other mutations

It is possible that other mutations that are unique to strain WRM31 but not part of the microdeletion could contribute to the observed phenotypes. Our recombination mapping and whole genome sequencing results identified 39 SNPs or indels as well as 2 larger deletions in addition to the microdeletion allele. While none of the SNPs or indels are found within the coding region of predicted or known protein-encoding genes, 10 are found within introns, 2 are found within predicted 3′UTRs, and 4 are found in regions upstream of genes. These mutations could conceivably affect the splicing, post-transcriptional regulation, or transcription of the associated genes, which has not been explicitly tested here. The 2 larger deletions disrupt genes of unknown function, *srj-38* and *K09C6.9*. It is unlikely that loss-of-function of either gene contributes to the observed phenotypes for reasons described above, but it remains possible that the mutations lead to truncated proteins that could have unexpected effects. Both mutations are tightly linked to the *sprDf1* microdeletion allele and cannot be easily segregated during outcrossing. More work will be necessary to define or rule out possible contributions from these mutations to the phenotypes observed.

### How did the microdeletion allele arise?

There are at least 2 potential explanations for how the microdeletion allele arose during our experiment. It is possible that one or more double strand DNA breaks were induced by Cas9 in our attempt to target the *mex-5* allele on chromosome IV. To assess the likelihood of this possibility, we used the program Cas-OFFinder to map potential off-target cleavage sites within the region removed in the microdeletion (Hwang *et al.* 2021). This analysis identified 3 weak off-target sites within the deleted region, each of which contains 5 mismatches to the guides with no more than 2 adjacent mismatches. None are near the precise breakpoints. It is, therefore, unlikely that any of these sites were efficiently targeted by the injected Cas9-guide RNA complex, but it cannot be ruled out.

The second possibility is that the allele arose spontaneously during our experiment before balancing. Indeed, there is evidence that this region of chromosome V may be prone to rearrangements. An overlapping segment of chromosome V (V:1,105,377–1,274,227) was found to be translocated to chromosome II in the divergent Hawaiian strain (CB4856) (Kim *et al.* 2019). Another overlapping region that removes an estimated 45 KB from chromosome V (V:855000..898999) was detected in a natural isolate from Australia (AB1) (Thompson *et al.* 2013). In microdeletion diseases, large deletions typically arise during meiosis through nonallelic homologous recombination, which can give rise to both duplications and deletions through mispairing of sister chromatids. No obvious homology is observed around the breakpoints of the *sprDf1* allele, suggesting that the microdeletion allele may not have arisen by this classic mechanism. However, extended microsatellite repeat sequences are located within a few 100 base pairs of both breakpoint sites (Zhang *et al.* 2022). An extended octonucleotide repeat sequence ATGCCTAC is found in 27 copies upstream of the left breakpoint, and 26 copies of a hexanucleotide repeat sequence CTAAGC are found upstream of the right breakpoint. Microsatellite repeat sequences are prone to expansion, mutation, and double-strand breaks caused by strand reannealing difficulties during DNA replication (Kim and Mirkin 2013; Gadgil *et al.* 2020). We suspect double stranded DNA breaks induced by difficulty in replicating the microsatellite repeats account for the large deletion.

Our work reinforces the importance of validating the genotype for alleles derived during CRISPR mutagenesis experiments. Directed sequencing methods that focus exclusively on predicted off target mutations could miss spontaneous mutations at unrelated loci, leading to misinterpretation. Given the low cost, we suggest routine resequencing of the *C. elegans* genome for alleles derived in this species. Outcrossing candidate mutants and generation of multiple lines where possible, can help mitigate this risk, and potentially reveal new mutants with interesting properties.

## Supplementary Material

jkad258_Supplementary_Data

## Data Availability

Strains and plasmids will be made available through the *Caenorhabditis Genetics Center* and Addgene, respectively. The authors confirm that all data are represented within the figures and tables of the manuscript and the supplementary files, with the exception of the genomic sequencing data described in Fig. 2. These data are available through the NCBI sequence read archive (SRA) at the following link: https://www.ncbi.nlm.nih.gov/sra/PRJNA899542. Supplemental material available at G3 online.
