# Supplementary material for: A nematode model to evaluate microdeletion phenotype expression
Source: G3 (Bethesda). 2023 Nov 13;14(2):jkad258. doi: 10.1093/g3journal/jkad258 (PMC10849325; doi:10.1093/g3journal/jkad258)
Supplement: jkad258_Supplementary_Data [file jkad258_supplementary_data.zip › suppl_data/Supplemental_Material_Legends_G3-2023-404629_Updated.docx]

**SUPPLEMENTARY MOVIE LEGENDS**

**Supplemental Movie 1**. Bursting Phenotype. The movie shows a time lapse of a GFP– *sprDf1* homozygous animal bursting. The time stamp is listed in the upper left hand corner, the video has been speed up to a rate of 4 frames per second for convenience.

**Supplemental Movie 2**. Uncoordinated Phenotype. The four panels show animals crawling on an unseeded NGM agar plate after being touched with a worm pick. The genotype of each panel is listed in the top left corner. The time stamp is listed in the bottom left corner. The frame rate is 4 frames per second and the movies are presented in real time.

**SUPPLEMENTAL FIGURE LEGENDS**

**Supplemental Figure 1**. Analysis of *srj-38* and *K09C6.9* deletions in WRM31 animals. A.Single worm PCR analysis of four phenotypic siblings reveal the presence of predicted deletions in *srj-38* (lanes 1-4) and *K09C6.9* (lanes 5-8). The expected band size is marked with a single asterisk. Interestingly, phenotypic animals also contained a full length band for *K09C6.9* (double asterisk), suggesting the phenotype isn’t caused by loss of function of this gene. The marker shown is New England Biolabs 1KB Plus (NEB, Ipswich, MA, Catalog Number N3200L). The Primers used are listed in Supplemental Table 3. B. The expression of *srj-38* mRNA is limited to dauer stage worms. The median FPKM data were recovered from Wormbase (<https://wormbase.org/species/c_elegans/gene/WBGene00005622#0-9fc1e2g4-10>) and are sourced from modEncode stage-specific RNA-seq libraries (Gerstein et al. 2010; Gerstein et al. 2014). C. Total and viable brood is presented comparing wild-type N2 animals to N2 animals treated with *srj-38* RNAi as described in the methods. Each point represents the brood of a single animal. The genotypes are listed below, and the black bar marks the mean of all animals scored. Statistical significance was assessed using a one-way ANOVA with Bonferroni correction for multiple hypothesis testing. There are no significant differences. D. Representative micrograph of of *srj-38* RNAi treated animals. There is no effect on embryo morphology or cytokinesis. Yellow arrows denote embryos. E. Treatment of N2 worms with *srj-38* RNAi does not induce bursting or reduced lifespan as assessed by Kaplan Meier Estimation using the logrank method. Error bars represent the standard error from the Kaplan Meier analysis. The *sprDf1/sprDf1* data from figure 4 is presented alongside for comparison in orange.

**Supplemental Figure 2**. The microdeletion mutation changes the embryo morphology. The length and width of each imaged embryo was determined from the images collected in D, and the aspect ratio (length / width) was also calculated for each embryo. The green dots represent the aspect ratio for embryos produced by GFP+ *sprDf1/nT1[qIs51]* hermaphrodites, and the blue dots represent embryos produced by GFP– *sprDf1/sprDf1* siblings. The black bars represent the mean, and statistical significance was assessed as by one way ANOVA with Bonferroni correction for multiple hypothesis testing across both days and genotypes. The p-values are reported between groups that show statistically significant differences.

**Supplemental Figure 3**. A. The overall length of GFP+ *sprDf1/nT1[qIs51]* heterozygotes and GFP– *sprDf1/sprDf1* homozygotes is shown as a function of days post hatching. Each point represents the length of a single animal. The black lines represent the mean. Statistical significance was assessed using a one-way ANOVA. Asterisks indicate a p-value < 0.05. B. A distension index, defined as the width at the vulva divided by the width at the pharynx, was calculated for each genotype from images collected as a function of days post hatching. The distribution, the mean, and statistical significance calculations are represented as in panel A. The abbreviation *nT1* is used in place of *nT1[qIs51]* throughout the figure.

**Reference:**

Gerstein MB, Lu ZJ, Van Nostrand EL, Cheng C, Arshinoff BI, Liu T, Yip KY, Robilotto R, Rechtsteiner A, Ikegami K, *et al*. 2010. Integrative analysis of the Caenorhabditis elegans genome by the modENCODE project. Science. 330(6012):1775–1787. doi:10.1126/ science.1196914.

Gerstein MB, Rozowsky J, Yan KK, Wang D, Cheng C, Brown JB, Davis CA, Hillier L, Sisu C, Li JJ, *et al*. 2014. Comparative analysis of the transcriptome across distant species. Nature. 512(7515):445–448. doi:10.1038/nature13424.
