# Supplementary material for: A nematode model to evaluate microdeletion phenotype expression
Source: G3 (Bethesda). 2023 Nov 13;14(2):jkad258. doi: 10.1093/g3journal/jkad258 (PMC10849325; doi:10.1093/g3journal/jkad258)
Supplement: jkad258_Supplementary_Data [file jkad258_supplementary_data.zip › suppl_data/Supplemental_Methods_G3-2023-404629/C202SC18110931_Caenorhabditis_elegans_Primary_Report/C202SC18110931_Caenorhabditis_elegans_Primary_Report.html]

 C202SC18110931\_Caenorhabditis\_elegans Resequencing Project Analysis Report 


- Library and Sequencing
  - DNA Qualification
  - Library Construction
  - Library Quality Control
  - DNA Sequencing
- Bioinformatics Pipeline
- Results of Analyses
  - Raw Data
  - Sequencing Data QC
  - Mapping
  - SNP Detection
  - InDel Detection
  - SV Detection
  - CNV Detection
  - Somatic SNP Detection & Annotation
  - Somatic InDel Detection & Annotation
- References
- Appendix
  - List of Softwares


# C202SC18110931\_Caenorhabditis\_elegans Resequencing Project Analysis Report

28-January-2019

- Library Preparation and Sequencing

- DNA Quantification
- Library Construction
- Library Quality Control
- High-throughput DNA Sequencing

- Bioinformatics Analysis Procedures
- Results of Analyses

- Raw Data
- Sequencing Data Quality Control

- Sequencing Quality Distribution
- Distribution of Sequencing Errors
- Sequencing Data Filtration
- Statistics of Sequencing Data
- Sequencing Evaluation Q&A

- Mapping Statistics

- Statistics of Reference Genome
- Mapping Statistics with Reference Genome
- Mapping Summary
- Mapping Q&A

- SNP Detection & Annotation

- Statistics of SNP Detection & Annotation
- SNP Quality Distribution
- SNP Mutation Frequency
- SNP Detection & Annotation Q&A

- InDel Detection & Annotation

- Statistics of InDel Detection & Annotation
- Length Distribution of CDS-located InDels
- InDel Detection & Annotation Q&A

- SV Detection & Annotation

- Statistics of SV Detection & Annotation
- Length Distribution of SVs
- SV Detection & Annotation Q&A

- CNV Detection & Annotation

- CNV Detection & Annotation Q&A

- Somatic SNP Detection & Annotation

- Statistics of Somatic SNP Detection & Annotation
- Somatic SNP Quality Distribution
- Somatic SNP Mutation Frequency

- Somatic InDel Detection & Annotation

- Statistics of Somatic InDel Detection & Annotation
- Length Distribution of CDS-located Somatic InDels

- References
- Appendix

- List of Softwares


Novogene Co., Ltd


---

  

## 1 Library Preparation and Sequencing

Throughout the whole process of sequencing from the DNA sample to the final data, each steps, including sample test, library preparation, and sequencing procedures, influences the quality of data production, while the data quality further impacts on the analysis results directly. To guarantee the accuracy and reliability of the sequencing data, Novogene utilizes stringent quality control (QC) procedures and strictly adheres to the high standard at each step from source. The workflow at Novogene is as follows:

Figure 1.1 Workflow of library preparation

### 1.1 DNA Qualification

Novogene utilizes three major QC methods for DNA sample qualification:

    (1) Agarose gel electrophoresis analysis for DNA purity and integrity;

    (2) Qubit® 2.0 flurometer quantitation for accurate measurement of DNA concentration;

Sample DNA with total amount of more than 1.5 ug was qualified for library construction.

  


### 1.2 Library Construction

The genomic DNA of each sample was randomly sheared into short fragments of about 350 bp, respectively. The obtained fragments were subjected to library construction using the NEBNext® DNA Library Prep Kit, with strictly following the instructions. Briefly, as followed by end repairing, dA-tailing, and further ligation with NEBNext adapter, the required fragments (in 300-500 bp size) were PCR enriched by P5 and indexed P7 oligos. After purification and subsequent quality check, the resulted library is ready for sequencing. The experimental procedures of DNA library preparation are shown in **Figure 1.2**.

Figure 1.2 Experimental procedures of library preparation

### 1.3 Library Quality Control

To check the prepared DNA libraries, Qubit® 2.0 fluorometer was firstly used to determine the concentration of the library. After dilution to 1 ng/ul, the Agilent® 2100 bioanalyzer was used to assess the insert size. And finally the quantitative real-time PCR (qPCR) was performed to detect the effective concentration of each library. If the library with appropriate insert size has an effective concentration of more than 2 nM, the constructed libraries are qualified and ready for Illumina® high-throughput sequencing.

### 1.4 High-throughput DNA Sequencing

The qualified DNA libraries were pooled according to their effective concentration as well as the expected data production. Pair-end sequencing were performed on Illumina® sequencing platform, with the read length of PE150 bp at each end.


Novogene Co., Ltd


---

  

## 2 Bioinformatics Analysis Procedures

The bioinformatic analysis procedures are as follows:

    (1) Quality control of raw sequencing data for clean data filtration;

    (2) Mapping clean reads to reference genome;

    (3) SNP, InDel, SV and CNV detection and annotation according to the reference genome mapping results.

Figure 2.1 Bioinformatics analysis pipeline


Novogene Co., Ltd


---

## 3 Results of Analyses

### 3.1 Raw Data

The original sequencing data acquired by high-throughput sequencing platforms (e.g. Illumina HiSeqTM /NovaSeqTM) recorded in image files are firstly transformed to sequence reads by base calling with the CASAVA software. The sequences and corresponding sequencing quality information are stored in a FASTQ file.

Every read in FASTQ format is stored in four lines as follows[1]:

@K00124:82:H2MH5BBXX:1:1101:31389:1158 2:N:0:0  
TAGCCACATAGAAACCAACAGCCATATAACTGGTAGCTTTAAGCGGCTCACCTTTAGCATCAACAGGCCACAACCAACCAGAACGTGAAAAAGCGTCCTGCGTGTAGCGAACTGCGATGGGCATACAGATCGGAAGAGCGTCGTGTAGGG  
+  
AAFFFKKKKKKKKFKKKFFKKAAFKKKKKFKKKKFKKA,FKKKKKKKKKAKKFKKKKKKKAKKKKKKFFKKKKF<FFKKKKKKKKKKKKKFKKFKKF7FFFFFKFKKKFKKKKKKKKF<FFKKKKFKKKKKFKFKFKKFK<<F,A7,AFK

Line 1 begins with an '@' character and is followed by Illumina sequence identifiers, and an optional description (such as a FASTA title line).  
Line 2 is the sequence of a sequencing read.  
Line 3 begins with a '+' character and is optionally followed by Illumina sequence identifier and description.  
Line 4 encodes the quality values for the sequence in Line 2, and must contain the same number of characters as the bases in the sequence. The per base sequencing quality score could be calculated by the ASCII value of each character in Line 4 minus a constant 33.

Table 3.1 Illumina sequence identifier details

|  |  |
| --- | --- |
| K00124 | Unique instrument name |
| 82 | Run ID |
| H2MH5BBXX | Flowcell ID |
| 1 | Flowcell lane |
| 1101 | Tile number within the flowcell lane |
| 31389 | 'x'-coordinate of the cluster within the tile |
| 1158 | 'y'-coordinate of the cluster within the tile |
| 2 | Member of a pair, 1 or 2 (paired-end or mate-pair reads only) |
| N | Y if the read fails filter (read is bad), N otherwise |
| 0 | 0 when none of the control bits are on, otherwise it is an even number |
| ATCACG | Index sequence |


Novogene Co., Ltd


---

### 3.2 Sequencing Data Quality Control

#### 3.2.1　Sequencing Quality Distribution

If the sequencing error rate is represented by e, and Illumina sequencing quality by Qphred, the quality score of a base (Phred score) is calculated by the following equation: Qphred=-10log10(e). The correspondence relationship between Illunima sequencing quality and Phred score in base calling by Casava version 1.8 is listed as follows:

Table 3.2 Sequencing error rate and corresponding base quality value

| Phred score | Error Rate | Correct Rate | Q-score |
| --- | --- | --- | --- |
| 10 | 1/10 | 90% | Q10 |
| 20 | 1/100 | 99% | Q20 |
| 30 | 1/1000 | 99.9% | Q30 |
| 40 | 1/10000 | 99.99% | Q40 |

For next-generation sequencing (NGS), the sequencing platform, chemical reactants, and sample quality can influence sequencing quality and base error rate. Sequencing quality distribution is examined over the full length of all sequences, to detect any sites (base positions) with an unusually low sequencing quality, where incorrect bases may be incorporated at abnormally high levels. For detailed sequencing quality distribution, please refer to **Figure 3.1**.

Figure 3.1 Distribution of sequencing quality

The x-axis shows the base position within a sequencing read, and the y-axis shows the average phred score of all reads at each position.  
(Pair-end sequencing data are plotted together, with the first PE150 bp representing read 1 and the following PE150 bp for read 2.)

Novogene Co., Ltd


---

  

#### 3.2.2 Distribution of Sequencing Errors

Sequencing error rate is related to the base quality of the obtained sequence. The sequencing platform, chemical reactants, and sample quality can all influence sequencing error rate and herein the base quality. For next-generation sequencing (NGS) with sequencing-by-synthesis strategy, sequencing error rate distribution shows two common features:

(1) Error rate increases with extending of the sequencing reads due to the consumption of chemical reagents, damage of the DNA template by laser irradiation, and possible accumulation of errors during the sequencing cycles. All the Illumina high-throughput sequencing platforms have this feature.  
(2) The sequencing error rate is higher for the first several bases than at other positions, which is likely the result of reading errors during the first few cycles after calibration of the optical instruments.

Sequencing error rate distribution is examined over the full length of all sequences, to detect any sites (base positions) with an unusually high error rate, where incorrect bases may be incorporated at abnormally high levels. For detailed sequencing error distribution, please refer to **Figure 3.2**.

  

Figure 3.2 Distribution of sequencing errors

The x-axis shows the base position within a sequencing read, and the y-axis shows the average error rate of all reads at each position.  
(Pair-end sequencing data are plotted together, with the first PE150 bp representing read 1 and the following PE150 bp for read 2.)

Novogene Co., Ltd


---

  

#### 3.2.3 Sequencing Data Filtration

Raw data obtained from sequencing contains adapter contamination and low-quality reads. These sequencing artifacts may increase the complexity of downstream analyses, and therefore, we utilize quality control steps to remove them. Consequently, all the downstream analyses are based on the clean reads. The quality control steps are as follows:

      (1) Discard the paired reads when either read contains adapter contamination;  
      (2) Discard the paired reads when uncertain nucleotides (N) constitute more than 10 percent of either read;  
      (3) Discard the paired reads when low quality nucleotides (base quality less than 5, Q ≤ 5) constitute more than 50 percent of either read.

Figure 3.3 Classification of the sequenced reads

(1) Adapter related: The proportion of filtered reads containing adapters in total reads.
(2) Containing N: The proportion of filtered reads containing more than 10% Ns in total reads.  
(3) Low quality: The proportion of filtered reads for low quality in total reads.
(4) Clean reads: The proportion of clean reads in raw reads.


Novogene Co., Ltd


---

  

#### 3.2.4 Statistics of Sequencing Data

Totally 8.1G raw data were sequenced from this run, with 8.1G clean data generated after filtering low-quality data, indicating the sufficient amount of data production. Statistics of sequencing data are listed in **Table 3.3**.

Table 3.3 Statistics of Sequencing Data

| Sample name | Lane | Raw reads | Raw data (G) | Clean data (G) | Effective (%) | Error (%) | Q20 (%) | Q30 (%) | GC (%) |
| --- | --- | --- | --- | --- | --- | --- | --- | --- | --- |
| VC362 | HGVYFDSXX\_L3 | 9321184 | 2.8 | 2.8 | 99.86 | 0.03 | 96.04 | 90.06 | 37.40 |
| N2 | HGVYFDSXX\_L3 | 10497465 | 3.1 | 3.1 | 99.83 | 0.03 | 96.34 | 90.64 | 36.11 |
| WRM31 | HGVYFDSXX\_L3 | 7206909 | 2.2 | 2.2 | 99.86 | 0.03 | 96.45 | 90.92 | 38.67 |

The details for the sequencing data statistics are as follows:

(1) Sample name: Sample name.  
(2) Lane: The flowcell ID and lane number during the sequencing (FlowcellID\_LaneNumber).  
(3) Raw reads: The number of sequencing reads pairs; four lines will be considered as one unit according to FASTQ format.   
(4) Raw data (G): The original sequence data volume.  
(5) Clean data (G): The sequence data volume calculated by clean data..  
(6) Effective (%): The ratio of clean data to raw data.  
(7) Error (%): Overall error rate of base.  
(8) Q20 (%): The percentage of bases with higher Phred score than 20.  
(9) Q30 (%): The percentage of bases with higher Phred score than 30.  
(10) GC: The percentage of G and C in the total bases.


Novogene Co., Ltd


---

  

#### 3.2.5 Sequencing Evaluation Q&A

|  |
| --- |
| **Q.:** As the sequencing error increases with the read length, what is the acceptable range of sequencing error rate? |
| **A.:** Currently for Illumina sequencers, the per base error rate is generally lower than 1%, and the highest acceptable threshold is 6%. |
| **Q.:** What is the data filtering criterion at Novogene? Is it strictly adhered? |
| **A.:** Novogene utilizes stringent data quality control procedures and strictly adheres to the high standard to guarantee the accuracy and reliability of the sequencing data. The detailed data quality control criterion is as follows:        **(1)** Discard the adapter-containing reads;        **(2)** Discard the paired reads when uncertain nucleotides (N) constitute more than 10 percent of either read;        **(3)** Discard the paired reads when low quality nucleotides (base quality less than 5, Q ≤ 5) constitute more than 50 percent of either read. |
| **List of Related Terms**: |
| **adapter**: the oligo nucleotides ligated to sample DNA at library preparation, for proper adhesion to the flow-cell via base-pairing in DNA sequencing. |
| **index**: the unique sequence tag for distinguishing each individual sample from multiplexing. |
| **Q20,Q30**: the proportion of bases with Phred score higher than 20 or 30. The Phred score, which is negatively correlated to the probability of incorrect base-calling, is calculated by the equation (Qphred=-10log10(e)) of sequencing error rate (e), indicating the sequencing quality. |
| **raw data/raw reads**: the original sequence data output by the instrument for a specific sample. |
| **clean data/clean reads**: the output of data from filtering raw data with quality control, which will be used for further analysis. |


Novogene Co., Ltd


---

Novogene Co., Ltd


---

  

#### 3.3.2　Mapping Statistics with Reference Genome

The mapping rates of samples reflect the similarity between each sample and the reference genome. The depth and coverage are indicators of the evenness and homology with the reference genome.

Table 3.5 Statistics of mapping rate, depth and coverage

| Sample | Mapped reads | Total reads | Mapping rate (%) | Average depth(X) | Coverage at least 1X (%) | Coverage at least 4X (%) | Duplicate (%) |
| --- | --- | --- | --- | --- | --- | --- | --- |
| N2 | 20,661,894 | 20,959,368 | 98.58 | 24.09 | 99.97 | 99.93 | 21.71 |
| VC362 | 17,092,757 | 18,616,474 | 91.82 | 20.61 | 99.97 | 99.91 | 19.0 |
| WRM31 | 12,488,373 | 14,393,426 | 86.76 | 15.50 | 99.96 | 99.75 | 16.61 |

The details for mapping statistics are as follows:

(1) Sample: Sample names.  
(2) Mapped reads: The number of clean reads mapped to the reference assembly, including both single-end reads and reads in pairs.  
(3) Total reads: Total number of effective reads in clean data.  
(4) Mapping rate: The ratio of the reference genome assembly mapped reads to the total sequenced clean reads.  
(5) Average depth: The average depth of mapped reads at each site, calculated by the total number of bases in the mapped reads dividing by size of the assembled genome.  
(6) Coverage at least 1X: The percentage of the assembled genome with more than one read at each site.  
(7) Coverage at least 4X: The percentage of the assembled genome with ≥4X coverage at each site.  
(8) Duplicate: The percentage of duplicated reads (percentage: duplicated reads/clean reads).

#### 3.3.3 Mapping Summary

For the current 100,286,401 bp reference genome, the mapping rate of each sample ranges from 86.76% to 98.58%. Refer to the reference genome (without Ns), the average depths are between 15.50X and 24.09X, and 1X coverages range from 99.96% to 99.97%. This result is in the qualified normal range and may serve in the subsequent variation detection and related analyses.

#### 3.3.4 Mapping Q&A

|  |
| --- |
| **Q.:** Which files are required for reference genome mapping? |
| **A.:** As for whole genome sequencing (WGS), only the corresponding reference genome file in FASTA format is required, while the whole exome sequencing (WES) also needs the target region file in BED format. |
| **Q.:** What are the potential causes of low mapping rate? |
| **A.:** The three major causes are: (1) with poorly assembled reference genome or a relatively far genetic relationship between the reference and the sample; (2) treatments to DNA that may changed the sequence (e.g. bisulfite treatment) and herein affecting the mapping rate and (3) contamination of other species. |
| **Q.:** Could the full-length reads be mapped to the reference? or should we trim from the sequence ends? |
| **A.:** According to the standard Illumina pair-end (PE) sequencing protocol, DNA fragments were ligated with adapters at both ends. The adapter habors universal sequences for flow-cell binding, DNA sequencing, as well as unique index located upstream of the sequencing primer for multiplexing. As for the PEPE150 sequencing of 350 bp library, the sequenced reads of PE150 bp at either end are adapter-free, which could be directly subjected to quality control for low quality reads filtration. The retaining sequences in PE150 bp length (namely clean data) are qualified for mapping with the reference genome. |

Novogene Co., Ltd


---

  

### 3.4 SNP Detection & Annotation

Single nucleotide polymorphism (SNP) refers to a variation in a single nucleotide which may occur at some specific position in the genome, including transition and transversion of a single nucleotide. We detected the individual SNP variations using GATK[4] with the following parameter: '-T HaplotypeCaller --gcpHMM 10 -stand\_emit\_conf 10 -stand\_call\_conf 30'.

To reduce the error rate in SNP detection, we filtered the results with the following parameter: 'QD < 2.0 || FS > 60.0 || MQ < 30.0 || HaplotypeScore > 13.0 || MappingQualityRankSum < -12.5 || eadPosRankSum < -8.0'.

#### 3.4.1 Statistics of SNP Detection & Annotation

ANNOVAR[5] is a widely used software in variation annotation with multiple capabilities, including gene-based annotation, region-based annotation, filter-based annotation as well as other functionalities. Novogene use ANNOVAR to do annotation of detected SNPs.

Table 3.6 Statistics of SNP detection and annotation

| Sample | Upstream | Exonic | | | | Intronic | Splicing | Downstream | Upstream/Downstream | Intergenic | ts | tv | ts/tv | Het rate(‰) | Total |
| --- | --- | --- | --- | --- | --- | --- | --- | --- | --- | --- | --- | --- | --- | --- | --- |
|  |  | Stop gain | Stop loss | Synonymous | Non-synonymous |  |  |  |  |  |  |  |  |  |  |
| N2 | 179 | 5 | 0 | 140 | 191 | 875 | 1 | 172 | 175 | 865 | 1362 | 1411 | 0.965 | 0.019 | 2773 |
| VC362 | 249 | 14 | 0 | 213 | 365 | 1094 | 5 | 255 | 268 | 936 | 2007 | 1626 | 1.234 | 0.027 | 3633 |
| WRM31 | 227 | 13 | 0 | 203 | 302 | 949 | 4 | 200 | 228 | 907 | 1742 | 1474 | 1.182 | 0.023 | 3216 |

The details for SNP detection and annotation statistics are as follows:

(1) Sample: Sample name;  
(2) Upstream: SNPs located within 1 kb upstream (away from transcription start site) of the gene.  
(3) Exonic: SNPs located in exonic region; Non-synonymous: single nucleotide mutation with changing amino acid sequence; Stop gain/loss: a nonsynonymous SNP that leads to the introduction/removal of stop codon at the variant site; Synonymous: single nucleotide mutation without changing amino acid sequence;  
(4) Intronic: SNPs located in intronic region;  
(5) Splicing: SNPs located in the splicing site (2 bp range of the intron/exon boundary).  
(6) Downstream: SNPs located within 1 kb downstream (away from transcription termination site) of the gene region.  
(7) Upstream/Downstream: SNPs located within the < 2 kb intergenic region, which is in 1 kb downstream or upstream of the genes.  
(8) Intergenic: SNPs located within the > 2 kb intergenic region.  
(9) ts: Transitions, a point mutation that changes a purine nucleotide to another purine (A ↔ G) or a pyrimidine nucleotide to another pyrimidine (C ↔ T). Approximately two out of three SNPs are transitions.  
(10) tv: Transversions, the substitution of a (two ring) purine for a (one ring) pyrimidine or *vice versa*.  
(11) ts/tv: The ratio of transitions to transversions.  
(12) Het rate: Genome-wide heterozygous rate, calculated by the ratio of heterozygous SNPs to the total number of genome bases.  
(13) Total: The total number of SNPs.

Novogene Co., Ltd


---

  

#### 3.4.2 SNP Quality Distribution

To assess the credibility of detected SNPs, we checked the distribution of support reads number, SNP quality, as well as the distance between adjacent SNPs. The results are shown in **Figure 3.4**.

Figure 3.4 Cumulative distribution of SNP quality

Note: These figures show the quality distribution of SNPs by, from top to bottom, the distribution of SNP support reads number, the distribution of distances between adjacent SNPs, and the cumulative distribution of SNP quality.

Novogene Co., Ltd


---

  

#### 3.4.3 SNP Mutation Frequency

Take the T:A>C:G mutations as an example, this category includes mutations from T to C and A to G. When T>C mutation appears on either of the double-strand, the A>G mutation will be found in the same position of the other chain. Therefore the T>C and A>G mutations are classified into one category. Accordingly, the whole-genome SNP mutations could be classified into six categories. The frequency of each type is shown in **Figure 3.5**.

Figure 3.5 Frequency of SNP mutations

The x-axis represents the number of the SNPs, and y-axis indicates the mutation types.

#### 3.4.4 SNP Detection & Annotation Q&A

|  |
| --- |
| **Q.:** What is the MQ quality for a SNP? |
| **A.:** The SNP quality is represented by the mapping quality of covering reads, calculated by the root-mean-square of the support reads' mapping quality. |
| **Q.:** What is QUAL value for a SNP? |
| **A.:** The QUAL value is the Phred quality score (QUAL), represents the probability (*p*) of SNP truly existing at certain position. The higher the QUAL value, the more likely the SNP exists. The relationship between QUAL and *p* is QUAL=-10\*log10 (1-*p*). Therefore, the QUAL value of 20 means the probability of the existence of this SNP is 99%. |
| **Q.:** What are transitions and transversions? |
| **A.:** Transitions refers to the changes between A and G, which are both purines, or between C and T, which are both pyrimidines; while transversions reprents changes between a purine and a pyrimidine, such as between A and T. |
| **Q.:** What is the heterozygous SNP? |
| **A.:** Heterozygous SNPs are those called with both REF (the same to reference) and ALT (different from reference) genotypes in a diploid species. |
| **Q.:** How to verify the SNP genotypes? |
| **A.:** The "golden standard" of SNP verification is PCR amplification followed by Sanger sequencing. |
| **Q.:** If the PCR-sequencing method failed to verify the detected SNP, did this mean that the NGS SNP calling is not reliable? |
| **A.:**  SNP calling in NGS is based on the support reads and depended on the sufficient coverage depth, which ensures the accuracy of most but not all the detected SNPs. We recommend to double-check the PCR results first, then use a genome browser such as *Savant* and *IGV* to manually check the mapped reads of the NGS result. |

Novogene Co., Ltd


---

  

### 3.5 InDel Detection & Annotation

InDel refers to the insertion or deletion of ≤ 50 bp sequences in the DNA. We detected the individual InDel variations using GATK[4] with the following parameter: '-T HaplotypeCaller --gcpHMM 10 -stand\_emit\_conf 10 -stand\_call\_conf 30'.

To reduce the error rate in InDel detection, we filtered the results with the following parameter: 'QD < 2.0 || FS > 200.0 || ReadPosRankSum < -20.0'.

#### 3.5.1 Statistics of InDel Detection & Annotation

Table 3.7 Statistics of InDel detection and annotation

| Sample | Upstream | Exonic | | | | | | Intronic | Splicing | Downstream | Upstream/Downstream | Intergenic | Insertion | Deletion | Het rate(‰) | Total |
| --- | --- | --- | --- | --- | --- | --- | --- | --- | --- | --- | --- | --- | --- | --- | --- | --- |
|  |  | Stop gain | Stop loss | Frameshift deletion | Frameshift insertion | Non-frameshift deletion | Non-frameshift insertion |  |  |  |  |  |  |  |  |  |
| N2 | 199 | 2 | 0 | 34 | 27 | 3 | 9 | 787 | 1 | 157 | 187 | 388 | 1138 | 813 | 0.008 | 1951 |
| VC362 | 200 | 2 | 0 | 38 | 31 | 7 | 8 | 819 | 1 | 186 | 218 | 357 | 1148 | 875 | 0.009 | 2023 |
| WRM31 | 202 | 1 | 0 | 38 | 30 | 10 | 7 | 702 | 1 | 160 | 175 | 363 | 1076 | 752 | 0.007 | 1829 |

The details of InDel annotation statistics are as follows:

(1) Sample: Sample names.  
(2) Upstream: InDels located within 1 kb upstream (away from transcription start site) of the gene.  
(3) Exonic: InDels located in exonic region; Stop gain/loss: InDel that leads to the introduction/removal of stop codon at the variant site; Frameshift deletion/insertion: InDel mutation changing the open reading frame with deletion or insertion; Non-Frameshift deletion/insertion:
InDel mutation without changing the open reading frame with deletion or insertion sequences of 3 or multiple of 3 bases;
  
(4) Intronic: InDel located in intronic region;  
(5) Splicing: InDel located in the splicing site (2 bp range of the intron/exon boundary).  
(6) Downstream: InDel located within 1 kb downstream (away from transcription termination site) of the gene region.  
(7) Upstream/Downstream: InDel SNPs located within the < 2 kb intergenic region, which is in 1 kb downstream or upstream of the genes.  
(8) Intergenic: InDel located within the > 2 kb intergenic region.  
(9) Het rate: InDel heterozygous rate, calculated by the ratio of InDels to the total number of genome bases.  
(10) Total: The total number of InDels.

Novogene Co., Ltd


---

  

#### 3.5.2 Length Distribution of CDS-located InDels

Figure 3.6 Length distribution of CDS-located InDels

The x-axis represents the proportion of the InDels with a certain length, and y-axis indicates the length of the InDels.

#### 3.5.3 InDel Detection & Annotation Q&A

|  |
| --- |
| **Q.:** What is the heterozygous rate for an InDel? |
| **A.:** Ratio of heterozygous InDels to total number of InDels, the heterozygous Indel is an Indel only located in one of the homologous chromosomes of a diploid sample. |
| **Q.:** What's the difference between an InDel and a SNP? |
| **A.:** SNP mutation refers to the change from one nucleotide to another nucleotide (eg. A↔T), while InDel is a type of mutation with insertion or deletion of one or more nucleotides (eg, A↔AT, with a T insertion). |

Novogene Co., Ltd


---

  

### 3.6 SV Detection & Annotation

Structural variants (SVs) are genomic variation with muations of relatively larger size (>50 bp), including deletions, duplications, insertions, inversions and translocations. BreakDancer[6] software were used to detect insertion (INS), deletion (DEL), inversion (INV), intra-chromosomal translocation (ITX) and inter-chromosomal translocation (CTX) mutations, based on the reference genome mapping results and the detected insert size. The detected SVs were filtered by removing those with less than 2 supporting PE reads, the INS, DEL and INV were further annotated by ANNOVAR.

#### 3.6.1 Statistics of SV Detection & Annotation

Table 3.8 Statistics of SV detection and annotation

| Sample | Upstream | Exonic | Downstream | Intronic | Upstream/Downstream | Intergenic | Splicing | INS | DEL | INV | ITX | CTX | Total |
| --- | --- | --- | --- | --- | --- | --- | --- | --- | --- | --- | --- | --- | --- |
| N2 | 8 | 49 | 17 | 22 | 8 | 80 | 0 | 6 | 149 | 42 | 851 | 76 | 1124 |
| VC362 | 13 | 53 | 10 | 13 | 8 | 53 | 0 | 2 | 118 | 43 | 799 | 85 | 1047 |
| WRM31 | 4 | 44 | 7 | 8 | 9 | 45 | 0 | 1 | 95 | 33 | 705 | 63 | 897 |

The details of SV detection statistics are as follows:

(1) Sample: Sample names.  
(2) Upstream: SVs located within 1 kb upstream (away from transcription start site) of the gene.  
(3) Exonic: SVs located in exonic region.  
(4) Intronic: SVs located in intronic region.  
(5) Downstream: SVs located within 1 kb downstream (away from transcription termination site) of the gene region.  
(6) Upstream/Downstream: SVs located within the < 2 kb intergenic region, which is in 1 kb downstream or upstream of the genes.  
(7) Intergenic: SVs located within the > 2 kb intergenic region.  
(8) Splicing: SVs located in the splicing site (2 bp range of the intron/exon boundary).  
(9) INS: Insersion.  
(10) DEL: Deletion.  
(11) INV: Inversion.  
(12) ITX: Intra-chromosomal translocations.  
(13) CTX: Inter-chromosomal translocations.  
(14) Total: The total number of SVs.

Novogene Co., Ltd


---

  

#### 3.6.2 Length Distribution of SVs

Figure 3.7 Length distribution of SVs

The x-axis represents samples, and the y-axis indicates the proportion of each type of SVs. Note, the length of DNA insert in library construction impacts the SVs detection greatly.

#### 3.6.3 SV Detection & Annotation Q&A

|  |
| --- |
| **Q.:** How did the SVs detected? |
| **A.:** There are four strategies for SV detection: (1) the read-pair technology, with detection of insertional or deletional mutations via aberrant insert size, and inversion via incorrect reading direction; (2) split-read approaches, which detects SVs with uncontinuously mapped reads on different positions of the reference genome; (3) the read-depth method, detecting CNVs caused by insertions and deletions; (4) *de novo* sequence assembly, with detection of SVs by comparison between the assembly and the reference genome. (For review, see *Alkan C, Coe BP, Eichler EE. Genome structural variation discovery and genotyping. Nature reviews Genetics. 2011;12(5):363-376.* from doi:10.1038/nrg2958.) Currently, the SV detection softwares are generally based on one of the principles, the Breakdancer software works with the read-pair method to detect the SVs. |
| **Q.:** Can Novogene provide the information of insertional or deletional fragments? Or, how could the SVs be verified? |
| **A.:** No, as limited by its read length and analysis, it's yet challenging for even the *de novo* assembling softwares with NGS data. The recommend strategy is to design specific primers and amplify the interested region and check the length variation on agarose gel or subject to Sanger sequencing. |
| **Q.:** Can Novogene provide us the flanking sequence adjacent to the mutation for designing primers? |
| **A.:** Novogene will provide the variant-related sequences on request. |

Novogene Co., Ltd


---

  

### 3.7 CNV Detection & Annotation

Copy-number variation (CNV) is a type of structual variation showing deletions or duplications in the genome. Based on the reads depth of the reference genome, CNVnator[7] were used to detect CNVs of potential deletions and duplications with the following parameter '-call 100'. The detected CNVs were further annotated by ANNOVAR.

Table 3.9 Statistics of CNV detection & annotation

| Sample | Upstream | Exonic | Intronic | Downstream | Upstream/Downstream | Intergenic | Duplication | Deletion | Duplication length (bp) | Deletion length (bp) | Total |
| --- | --- | --- | --- | --- | --- | --- | --- | --- | --- | --- | --- |
| N2 | 7 | 45 | 11 | 12 | 4 | 31 | 86 | 33 | 499600 | 250100 | 119 |
| VC362 | 5 | 56 | 4 | 12 | 3 | 29 | 93 | 25 | 718800 | 276300 | 118 |
| WRM31 | 4 | 53 | 4 | 8 | 2 | 25 | 81 | 22 | 733500 | 327600 | 103 |

The details of CNV detection and annotation are as follows:

(1) Sample: Sample names.  
(2) Upstream: CNVs located within 1 kb upstream (away from transcription start site) of the gene.  
(3) Exonic: CNVs located in exonic region.  
(4) Intronic: CNVs located in intronic region.  
(5) Downstream: CNVs located within 1 kb downstream (away from transcription termination site) of the gene region.  
(6) Upstream/Downstream: CNVs located within the < 2 kb intergenic region, which is in 1 kb downstream or upstream of the genes.  
(7) Intergenic: CNVs located within the > 2 kb intergenic region.  
(8) Duplication: CNVs with increased copy number.  
(9) Deletion: CNVs with decreased copy number.  
(10) Duplication length (bp): The total length of CNV duplication.  
(11) Deletion length (bp): The total length of CNV deletion.  
(12) Total: The total number of CNVs.

Figure 3.8 CNV annotation

The distribution of CNVs on the genome.

#### 3.7.1 CNV Detection & Annotation Q&A

|  |
| --- |
| **Q.:** What is the strategy for CNV detection? |
| **A.:** In brief, CNVs are called using read depths of bins, with involving GC calibration and sequencing uniformity calibration. |
| **Q.:** What is the original copy number? |
| **A.:** For haploid, the original copy number is 1; while for diploid, the original copy number is 2. |

Novogene Co., Ltd


---

  


### 3.9 Somatic SNP Detection & Annotation

We detected the individual SNP variations using GATK[4] with the following parameter: '-T MuTect2'.

#### 3.9.1 Statistics of Somatic SNP Detection & Annotation

ANNOVAR[5] is a widely used software in variation annotation with multiple capabilities, including gene-based annotation, region-based annotation, filter-based annotation as well as other functionalities. Novogene use ANNOVAR to do annotation of detected somatic SNPs.

Table 3.10 Statistics of Somatic SNP detection and annotation

| Group | Upstream | Exonic | | | | Intronic | Splicing | Downstream | Upstream/Downstream | Intergenic | ts | tv | ts/tv | Het rate(%) | Total |
| --- | --- | --- | --- | --- | --- | --- | --- | --- | --- | --- | --- | --- | --- | --- | --- |
|  |  | Stop gain | Stop loss | Synonymous | Non-synonymous |  |  |  |  |  |  |  |  |  |  |
| N2\_WRM31 | 65 | 12 | 0 | 73 | 161 | 255 | 5 | 52 | 101 | 178 | 623 | 349 | 1.785 | 0.010 | 972 |
| VC362\_WRM31 | 32 | 6 | 0 | 29 | 48 | 100 | 2 | 24 | 22 | 112 | 233 | 209 | 1.115 | 0.004 | 442 |

The details for somatic SNP detection and annotation statistics are as follows:

(1) Sample: Sample name;  
(2) Upstream: SNPs located within 1 kb upstream (away from transcription start site) of the gene.  
(3) Exonic: SNPs located in exonic region; Non-synonymous: single nucleotide mutation with changing amino acid sequence; Stop gain/loss: a nonsynonymous SNP that leads to the introduction/removal of stop codon at the variant site; Synonymous: single nucleotide mutation without changing amino acid sequence;  
(4) Intronic: SNPs located in intronic region;  
(5) Splicing: SNPs located in the splicing site (2 bp range of the intron/exon boundary).  
(6) Downstream: SNPs located within 1 kb downstream (away from transcription termination site) of the gene region.  
(7) Upstream/Downstream: SNPs located within the < 2 kb intergenic region, which is in 1 kb downstream or upstream of the genes.  
(8) Intergenic: SNPs located within the > 2 kb intergenic region.  
(9) ts: Transitions, a point mutation that changes a purine nucleotide to another purine (A ↔ G) or a pyrimidine nucleotide to another pyrimidine (C ↔ T). Approximately two out of three SNPs are transitions.  
(10) tv: Transversions, the substitution of a (two ring) purine for a (one ring) pyrimidine or *vice versa*.  
(11) ts/tv: The ratio of transitions to transversions.  
(12) Het rate: Genome-wide heterozygous rate, calculated by the ratio of heterozygous SNPs to the total number of genome bases.  
(13) Total: The total number of SNPs.

Novogene Co., Ltd


---

  


#### 3.9.2 Somatic SNP Quality Distribution

To assess the credibility of detected somatic SNPs, we checked the distribution of support reads number and the distance between adjacent SNPs. The results are shown in **Figure 3.10**.

Figure 3.10 Cumulative distribution of somatic SNP quality

Note: These figures show the quality distribution of SNPs by, from top to bottom, the distribution of SNP support reads number and the distribution of distances between adjacent SNPs.

Novogene Co., Ltd


---

  

#### 3.9.3 Somatic SNP Mutation Frequency

Take the T:A>C:G mutations as an example, this category includes mutations from T to C and A to G. When T>C mutation appears on either of the double-strand, the A>G mutation will be found in the same position of the other chain. Therefore the T>C and A>G mutations are classified into one category. Accordingly, the whole-genome somatic SNP mutations could be classified into six categories. The frequency of each type is shown in **Figure 3.11**.

Figure 3.11 Frequency of somatic SNP mutations

The x-axis represents the number of the somatic SNPs, and y-axis indicates the mutation types.


Novogene Co., Ltd


---

  

### 3.10 Somatic InDel Detection & Annotation

We detected the individual SNP variations using GATK[4] with the following parameter: '-T MuTect2'.

#### 3.10.1 Statistics of Somatic InDel Detection & Annotation

Table 3.11 Statistics of somatic InDel detection and annotation

| Group | Upstream | Exonic | | | | | | Intronic | Splicing | Downstream | Upstream/Downstream | Intergenic | Insertion | Deletion | Het rate(%) | Total |
| --- | --- | --- | --- | --- | --- | --- | --- | --- | --- | --- | --- | --- | --- | --- | --- | --- |
|  |  | Stop gain | Stop loss | Frameshift deletion | Frameshift insertion | Non-frameshift deletion | Non-frameshift insertion |  |  |  |  |  |  |  |  |  |
| N2\_WRM31 | 23 | 0 | 0 | 8 | 5 | 13 | 0 | 58 | 0 | 17 | 29 | 36 | 53 | 158 | 0.002 | 211 |
| VC362\_WRM31 | 12 | 0 | 0 | 6 | 3 | 9 | 0 | 43 | 0 | 14 | 11 | 24 | 27 | 120 | 0.001 | 147 |

The details of somatic InDel annotation statistics are as follows:

(1) Sample: Sample names.  
(2) Upstream: InDels located within 1 kb upstream (away from transcription start site) of the gene.  
(3) Exonic: InDels located in exonic region; Stop gain/loss: InDel that leads to the introduction/removal of stop codon at the variant site; Frameshift deletion/insertion: InDel mutation changing the open reading frame with deletion or insertion; Non-Frameshift deletion/insertion:
InDel mutation without changing the open reading frame with deletion or insertion sequences of 3 or multiple of 3 bases;
  
(4) Intronic: InDel located in intronic region;  
(5) Splicing: InDel located in the splicing site (2 bp range of the intron/exon boundary).  
(6) Downstream: InDel located within 1 kb downstream (away from transcription termination site) of the gene region.  
(7) Upstream/Downstream: InDel SNPs located within the < 2 kb intergenic region, which is in 1 kb downstream or upstream of the genes.  
(8) Intergenic: InDel located within the > 2 kb intergenic region.  
(9) Het rate: InDel heterozygous rate, calculated by the ratio of InDels to the total number of genome bases.  
(10) Total: The total number of InDels.

Novogene Co., Ltd


---

  

#### 3.10.2 Length Distribution of CDS-located Somatic InDels

Figure 3.12 Length distribution of CDS-located Somatic InDels

The x-axis represents the proportion of the somatic InDels with a certain length, and y-axis indicates the length of the somatic InDels.


Novogene Co., Ltd


---

  

## 4 References

[1] Cock, P.J.A., Fields, et al. The Sanger FASTQ file format for sequences with quality scores, and the Solexa/Illumina FASTQ variants. Nucleic Acids Research. 2010, 38(6):1767-1771.

[2] Li H, Durbin R. Fast and accurate short read alignment with Burrows-Wheeler transform. Bioinformatics. 2009, 25(14):1754-1760.

[3] Li H, Handsaker B, Wysoker A, et al. The Sequence Alignment/Map format and SAMtools. Bioinformatics. 2009, 25(16):2078-2079.

[4] DePristo M A, Banks E, Poplin R, et al. A framework for variation discovery and genotyping using next-generation DNA sequencing data. Nature Genetics. 2011, 43(5):491-498.

[5] Wang K, Li M, Hakonarson H. ANNOVAR: functional annotation of genetic variants from high-throughput sequencing data. Nucleic Acids Research. 2010, 38(16):e164.

[6] Chen, K, et al. BreakDancer: an algorithm for high-resolution mapping of genomic structural variation. Nature Methods. 2009, 6:677-681.

[7] Abyzov A, Urban A E, Snyder M, et al. CNVnator: an approach to discover, genotype, and characterize typical and atypical CNVs from family and population genome sequencing. Genome research. 2011, 21(6):974-984.

[8] Krzywinski M, Schein J, Birol I, et al. Circos: an information aesthetic for comparative genomics. Genome Research. 2009, 19(9):1639-1645.

Novogene Co., Ltd


---

  

## 5 Appendix

### 5.1 List of Softwares

Novogene provides the software list in bioinformatics analysis pipeline for your reference.

Table 5.1 List of softwares in WGS analyses

| Analysis | Software | Usage | Version |
| --- | --- | --- | --- |
| Quality control | in-house | Quality control | 1.0 |
| Mapping | BWA | Mapping clean reads to the reference genome and generation of bam result files. | 0.7.8-r455 |
|  | SAMtools | Sorting the bam files and removing duplication reads. | 0.1.19-44428cd |
|  | Picard | Merging the bam files from the same sample. | 1.111 |
| SNP/InDel Detection | GATK | Detection and filtration of SNPs and InDels. | v3.5 |
| SV Detection | Breakdancer | SV detection. | 1.4.4 |
| CNV Detection | CNVnator | CNV detection. | V0.3 |
| Somatic SNP/InDel Detection | GATK | Detection and filtration of somatic SNPs and InDels. | v3.5 |
| Variation Annotation | ANNOVAR | Annotation of the detected variations. | 2015Mar22 |
