# Supplementary figures and images for: A nematode model to evaluate microdeletion phenotype expression
Source: G3 (Bethesda). 2023 Nov 13;14(2):jkad258. doi: 10.1093/g3journal/jkad258 (PMC10849325; doi:10.1093/g3journal/jkad258)

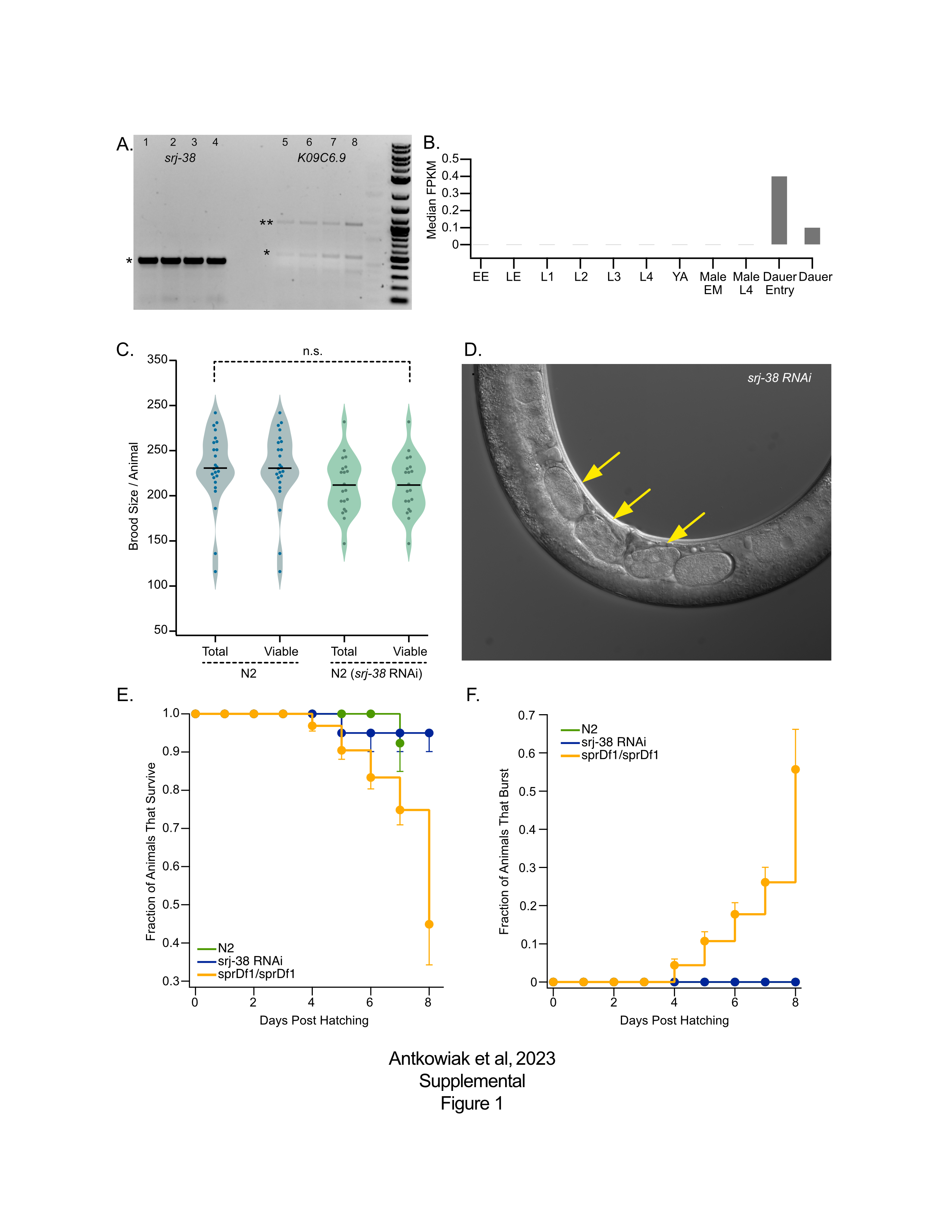

Supplement: jkad258_Supplementary_Data [file jkad258_supplementary_data.zip › suppl_data/Supplemental_Figure_1_G3-2023-404629.tif]

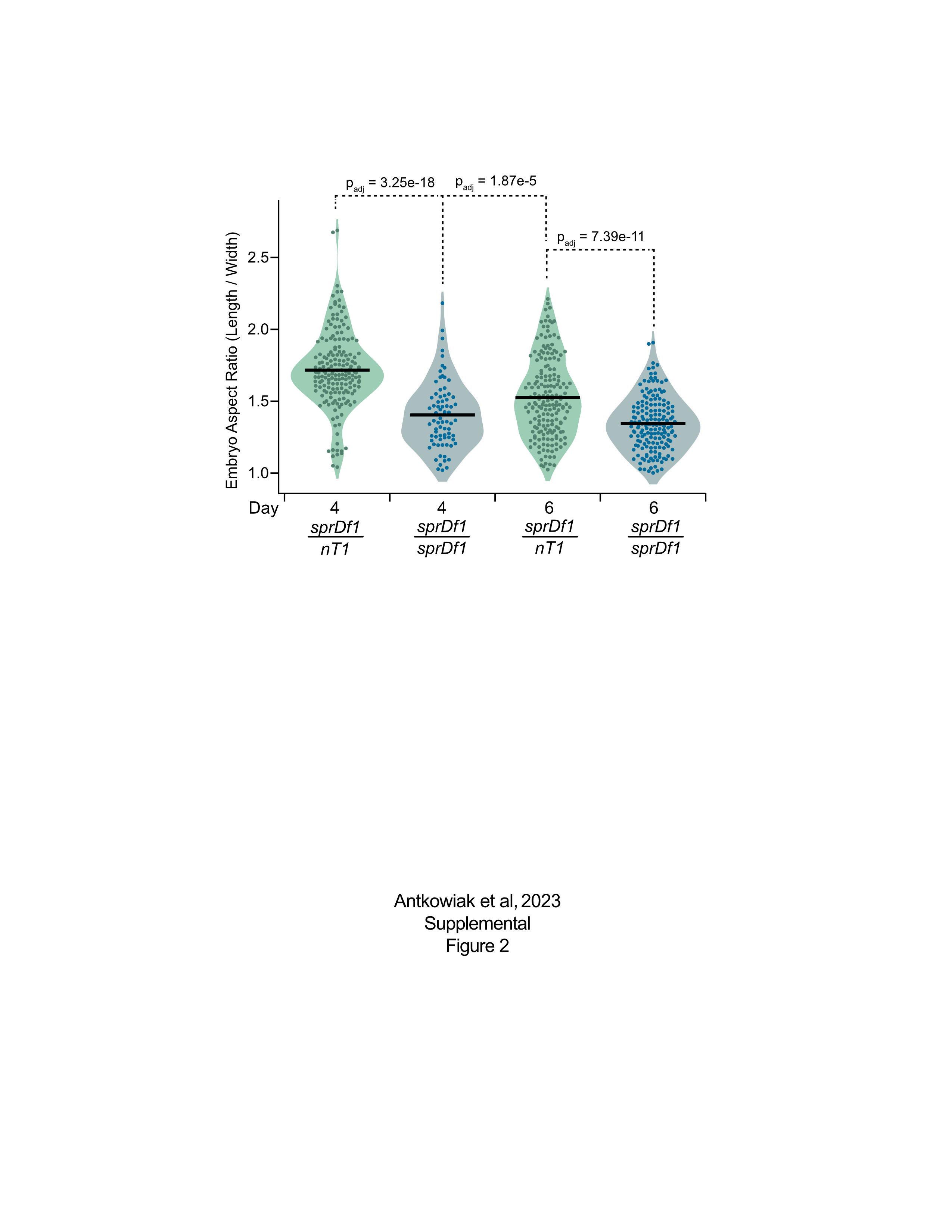

Supplement: jkad258_Supplementary_Data [file jkad258_supplementary_data.zip › suppl_data/Supplemental_Figure_2_G3-2023-404629.tif]

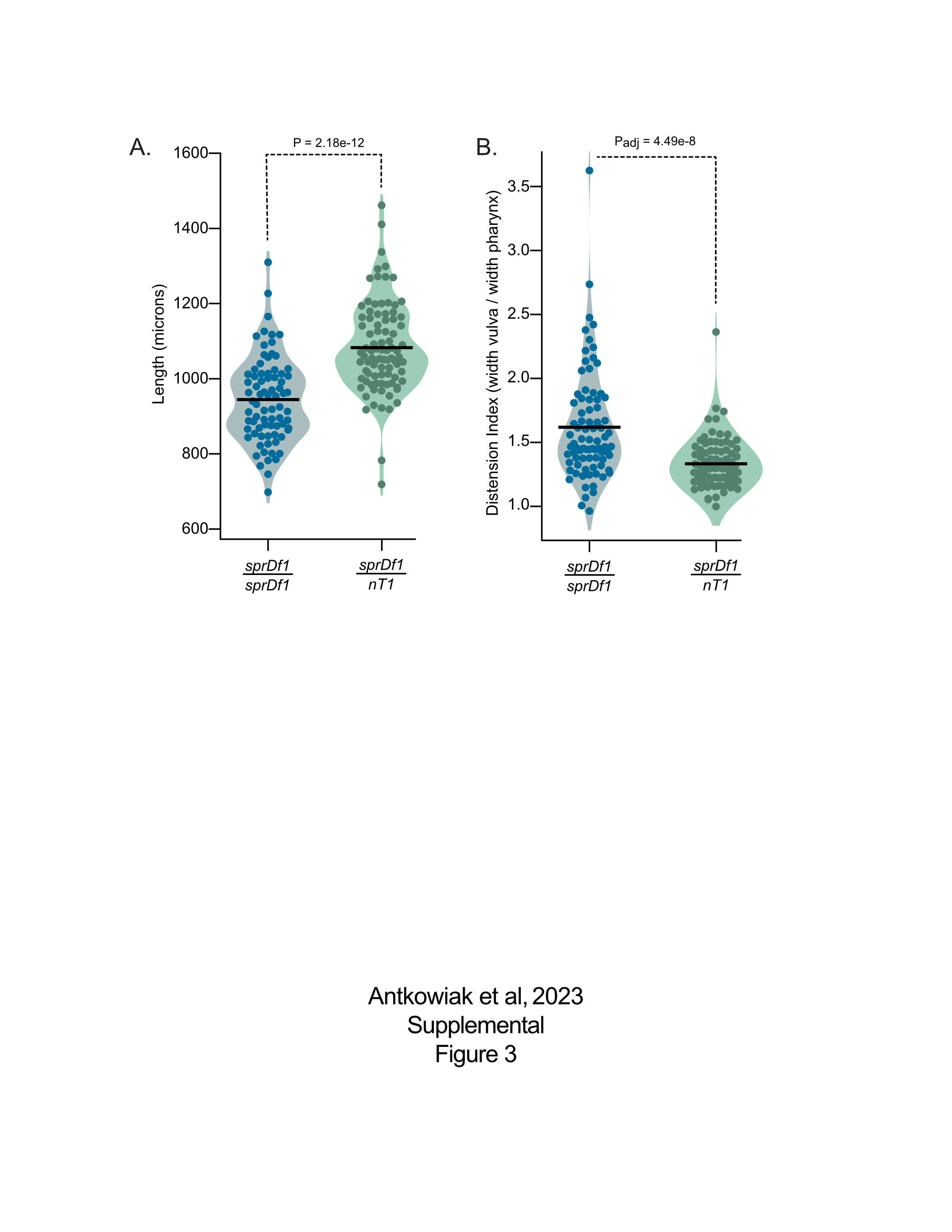

Supplement: jkad258_Supplementary_Data [file jkad258_supplementary_data.zip › suppl_data/Supplemental_Figure_3_G3-2023-404629.tif]

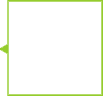

Supplement: jkad258_Supplementary_Data [file jkad258_supplementary_data.zip › suppl_data/Supplemental_Methods_G3-2023-404629/C202SC18110931_Caenorhabditis_elegans_Primary_Report/src/images/album-slider-arrow_box.png]

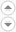

Supplement: jkad258_Supplementary_Data [file jkad258_supplementary_data.zip › suppl_data/Supplemental_Methods_G3-2023-404629/C202SC18110931_Caenorhabditis_elegans_Primary_Report/src/images/album-slider-button.png]

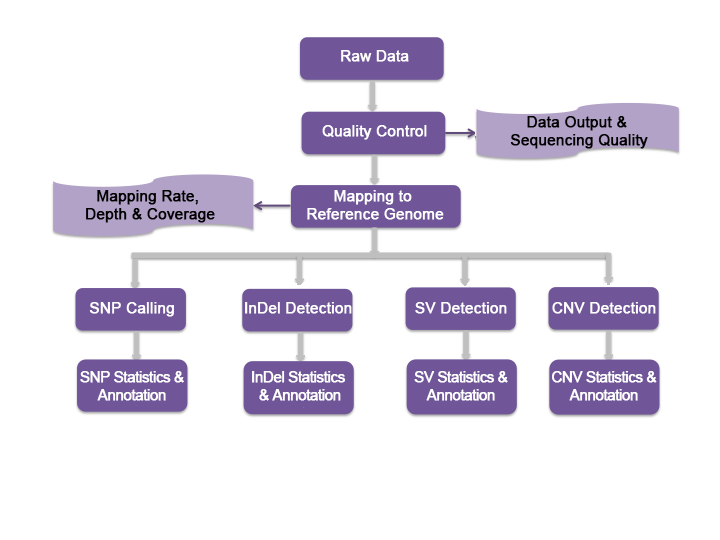

Supplement: jkad258_Supplementary_Data [file jkad258_supplementary_data.zip › suppl_data/Supplemental_Methods_G3-2023-404629/C202SC18110931_Caenorhabditis_elegans_Primary_Report/src/images/bioinfoWorkflow.png]

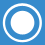

Supplement: jkad258_Supplementary_Data [file jkad258_supplementary_data.zip › suppl_data/Supplemental_Methods_G3-2023-404629/C202SC18110931_Caenorhabditis_elegans_Primary_Report/src/images/close.gif]

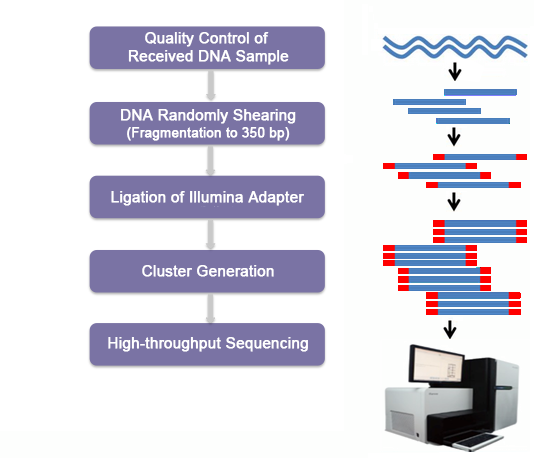

Supplement: jkad258_Supplementary_Data [file jkad258_supplementary_data.zip › suppl_data/Supplemental_Methods_G3-2023-404629/C202SC18110931_Caenorhabditis_elegans_Primary_Report/src/images/experimentWorkflow.png]

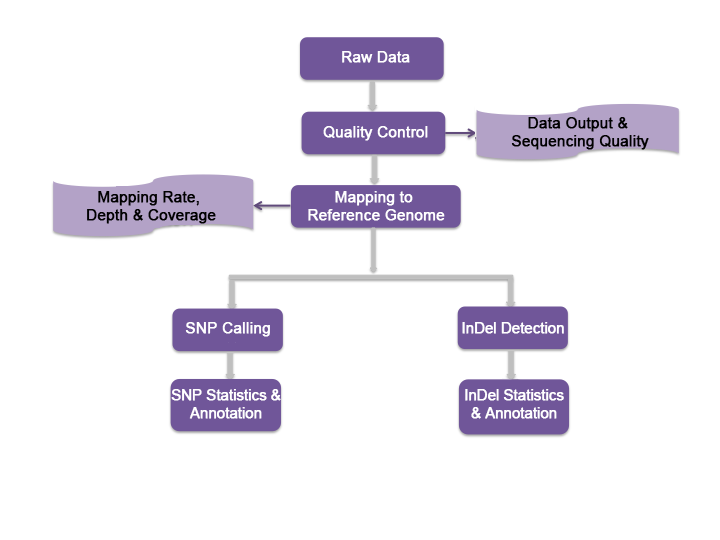

Supplement: jkad258_Supplementary_Data [file jkad258_supplementary_data.zip › suppl_data/Supplemental_Methods_G3-2023-404629/C202SC18110931_Caenorhabditis_elegans_Primary_Report/src/images/flow_snpindel.png]

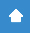

Supplement: jkad258_Supplementary_Data [file jkad258_supplementary_data.zip › suppl_data/Supplemental_Methods_G3-2023-404629/C202SC18110931_Caenorhabditis_elegans_Primary_Report/src/images/goTop.jpg]

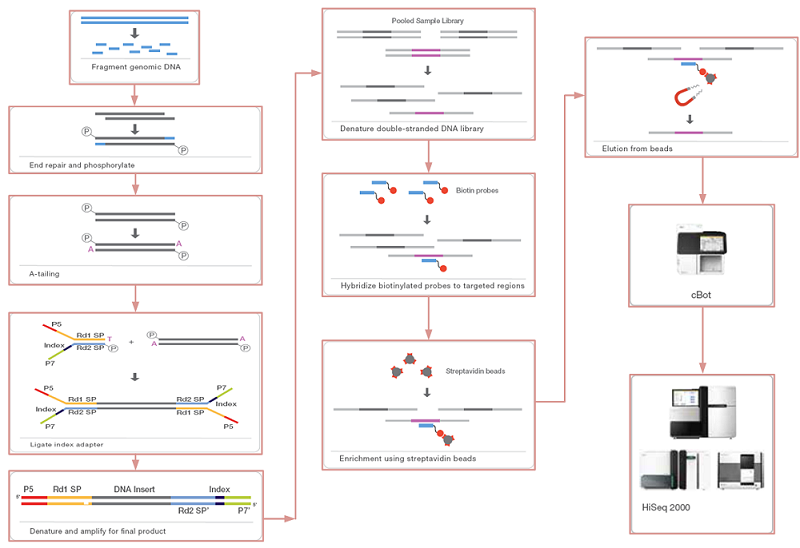

Supplement: jkad258_Supplementary_Data [file jkad258_supplementary_data.zip › suppl_data/Supplemental_Methods_G3-2023-404629/C202SC18110931_Caenorhabditis_elegans_Primary_Report/src/images/illumina_experiment_pipeline.png]

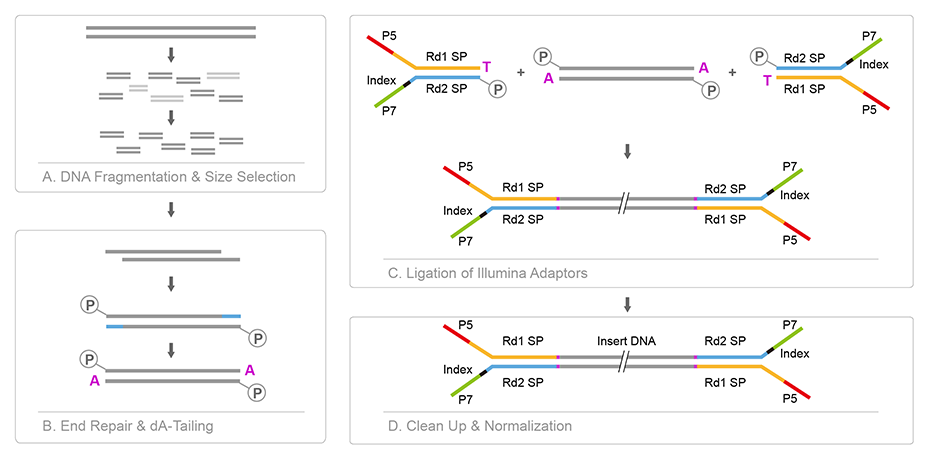

Supplement: jkad258_Supplementary_Data [file jkad258_supplementary_data.zip › suppl_data/Supplemental_Methods_G3-2023-404629/C202SC18110931_Caenorhabditis_elegans_Primary_Report/src/images/LibPrep_PCR-free.png]

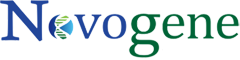

Supplement: jkad258_Supplementary_Data [file jkad258_supplementary_data.zip › suppl_data/Supplemental_Methods_G3-2023-404629/C202SC18110931_Caenorhabditis_elegans_Primary_Report/src/images/logo.png]

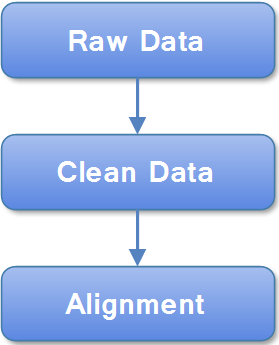

Supplement: jkad258_Supplementary_Data [file jkad258_supplementary_data.zip › suppl_data/Supplemental_Methods_G3-2023-404629/C202SC18110931_Caenorhabditis_elegans_Primary_Report/src/images/mapping.png]

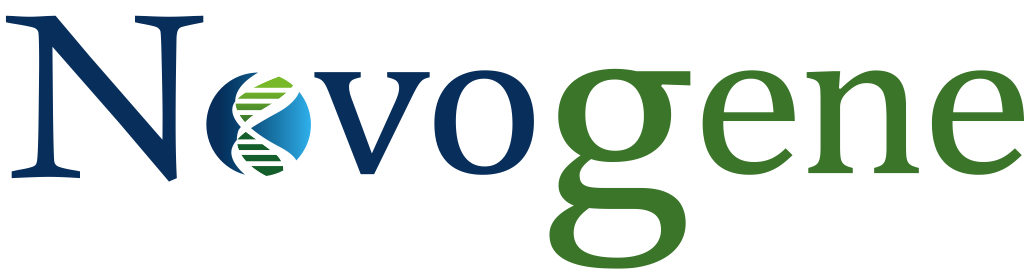

Supplement: jkad258_Supplementary_Data [file jkad258_supplementary_data.zip › suppl_data/Supplemental_Methods_G3-2023-404629/C202SC18110931_Caenorhabditis_elegans_Primary_Report/src/images/novogeneLogo.png]

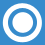

Supplement: jkad258_Supplementary_Data [file jkad258_supplementary_data.zip › suppl_data/Supplemental_Methods_G3-2023-404629/C202SC18110931_Caenorhabditis_elegans_Primary_Report/src/images/open.gif]

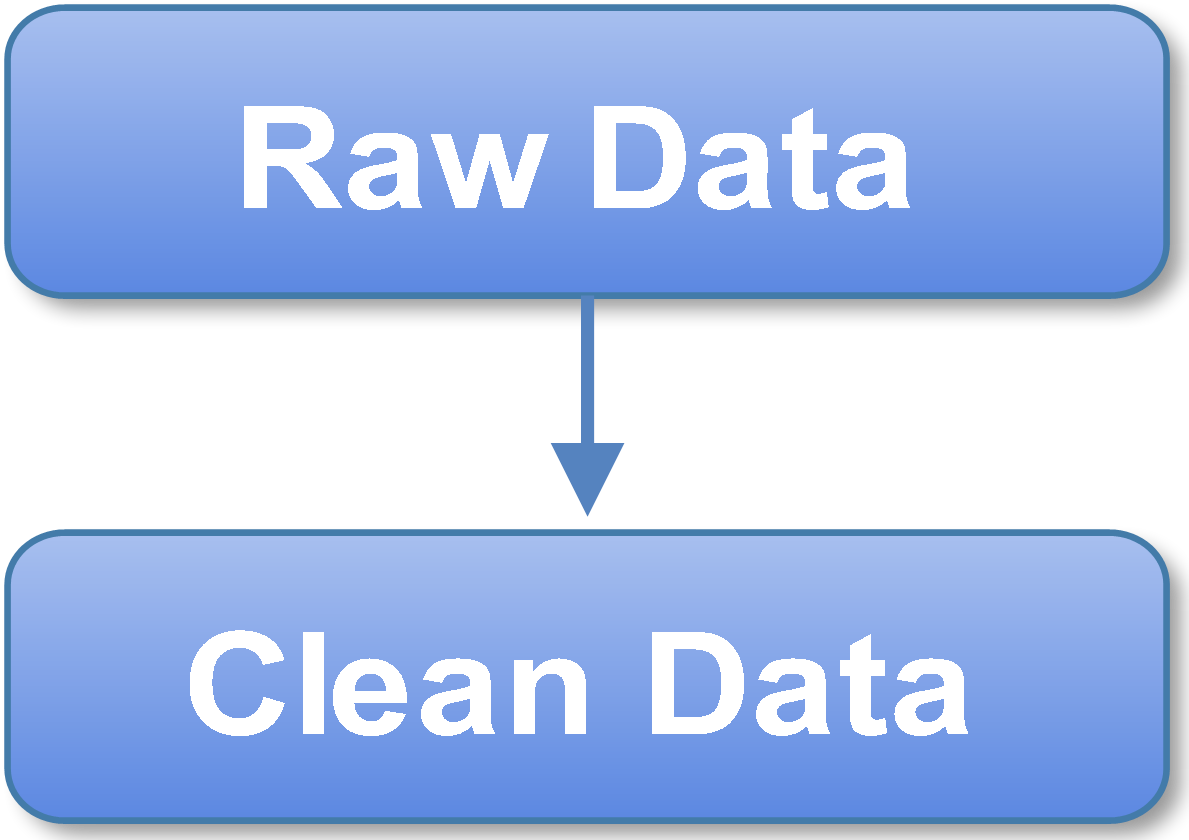

Supplement: jkad258_Supplementary_Data [file jkad258_supplementary_data.zip › suppl_data/Supplemental_Methods_G3-2023-404629/C202SC18110931_Caenorhabditis_elegans_Primary_Report/src/images/QC.png]

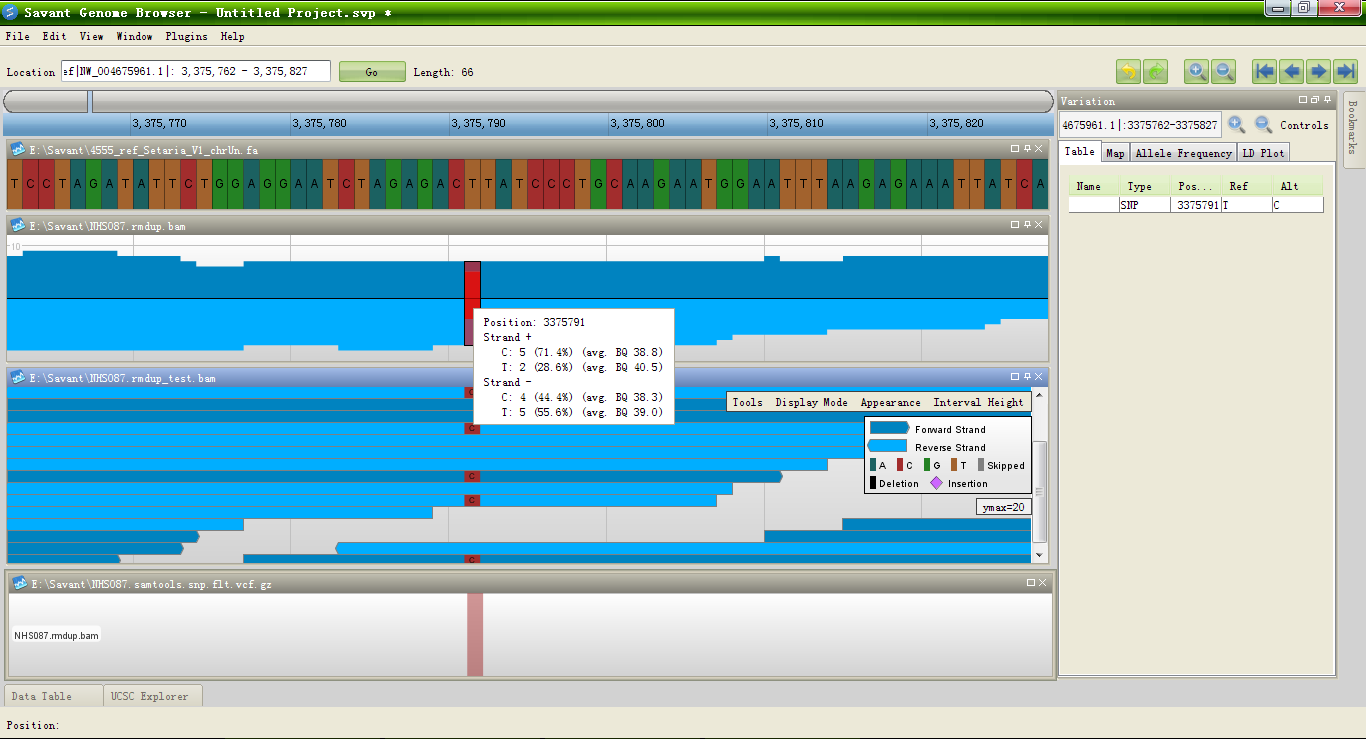

Supplement: jkad258_Supplementary_Data [file jkad258_supplementary_data.zip › suppl_data/Supplemental_Methods_G3-2023-404629/C202SC18110931_Caenorhabditis_elegans_Primary_Report/src/images/savant.png]

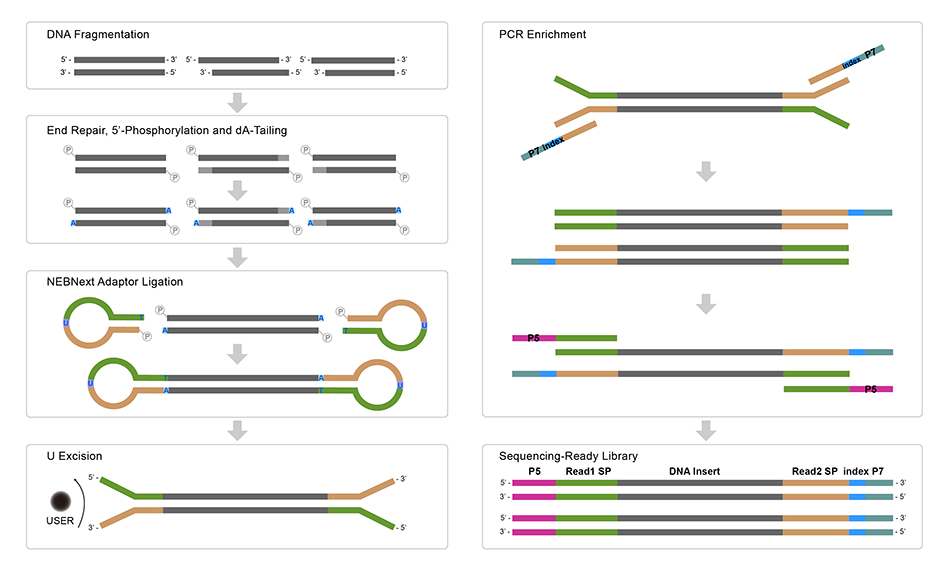

Supplement: jkad258_Supplementary_Data [file jkad258_supplementary_data.zip › suppl_data/Supplemental_Methods_G3-2023-404629/C202SC18110931_Caenorhabditis_elegans_Primary_Report/src/images/wgs_experiment_pipeline.png]

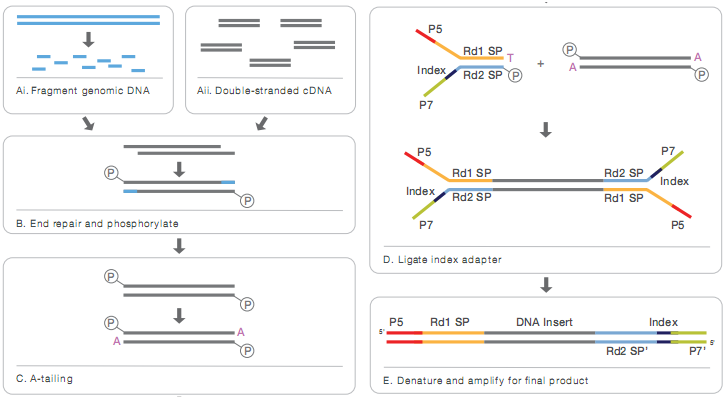

Supplement: jkad258_Supplementary_Data [file jkad258_supplementary_data.zip › suppl_data/Supplemental_Methods_G3-2023-404629/C202SC18110931_Caenorhabditis_elegans_Primary_Report/src/images/wgs_experiment_pipeline_before.png]

Supplement: jkad258_Supplementary_Data [file jkad258_supplementary_data.zip › suppl_data/Supplemental_Methods_G3-2023-404629/C202SC18110931_Caenorhabditis_elegans_Primary_Report/src/js/fancybox/blank.gif]

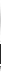

Supplement: jkad258_Supplementary_Data [file jkad258_supplementary_data.zip › suppl_data/Supplemental_Methods_G3-2023-404629/C202SC18110931_Caenorhabditis_elegans_Primary_Report/src/js/fancybox/fancybox-x.png]

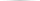

Supplement: jkad258_Supplementary_Data [file jkad258_supplementary_data.zip › suppl_data/Supplemental_Methods_G3-2023-404629/C202SC18110931_Caenorhabditis_elegans_Primary_Report/src/js/fancybox/fancybox-y.png]

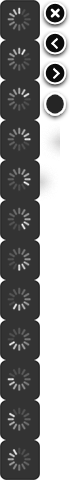

Supplement: jkad258_Supplementary_Data [file jkad258_supplementary_data.zip › suppl_data/Supplemental_Methods_G3-2023-404629/C202SC18110931_Caenorhabditis_elegans_Primary_Report/src/js/fancybox/fancybox.png]

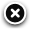

Supplement: jkad258_Supplementary_Data [file jkad258_supplementary_data.zip › suppl_data/Supplemental_Methods_G3-2023-404629/C202SC18110931_Caenorhabditis_elegans_Primary_Report/src/js/fancybox/fancy_close.png]

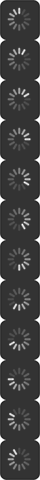

Supplement: jkad258_Supplementary_Data [file jkad258_supplementary_data.zip › suppl_data/Supplemental_Methods_G3-2023-404629/C202SC18110931_Caenorhabditis_elegans_Primary_Report/src/js/fancybox/fancy_loading.png]

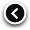

Supplement: jkad258_Supplementary_Data [file jkad258_supplementary_data.zip › suppl_data/Supplemental_Methods_G3-2023-404629/C202SC18110931_Caenorhabditis_elegans_Primary_Report/src/js/fancybox/fancy_nav_left.png]

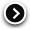

Supplement: jkad258_Supplementary_Data [file jkad258_supplementary_data.zip › suppl_data/Supplemental_Methods_G3-2023-404629/C202SC18110931_Caenorhabditis_elegans_Primary_Report/src/js/fancybox/fancy_nav_right.png]

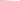

Supplement: jkad258_Supplementary_Data [file jkad258_supplementary_data.zip › suppl_data/Supplemental_Methods_G3-2023-404629/C202SC18110931_Caenorhabditis_elegans_Primary_Report/src/js/fancybox/fancy_shadow_e.png]

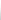

Supplement: jkad258_Supplementary_Data [file jkad258_supplementary_data.zip › suppl_data/Supplemental_Methods_G3-2023-404629/C202SC18110931_Caenorhabditis_elegans_Primary_Report/src/js/fancybox/fancy_shadow_n.png]

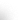

Supplement: jkad258_Supplementary_Data [file jkad258_supplementary_data.zip › suppl_data/Supplemental_Methods_G3-2023-404629/C202SC18110931_Caenorhabditis_elegans_Primary_Report/src/js/fancybox/fancy_shadow_ne.png]

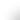

Supplement: jkad258_Supplementary_Data [file jkad258_supplementary_data.zip › suppl_data/Supplemental_Methods_G3-2023-404629/C202SC18110931_Caenorhabditis_elegans_Primary_Report/src/js/fancybox/fancy_shadow_nw.png]

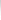

Supplement: jkad258_Supplementary_Data [file jkad258_supplementary_data.zip › suppl_data/Supplemental_Methods_G3-2023-404629/C202SC18110931_Caenorhabditis_elegans_Primary_Report/src/js/fancybox/fancy_shadow_s.png]

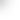

Supplement: jkad258_Supplementary_Data [file jkad258_supplementary_data.zip › suppl_data/Supplemental_Methods_G3-2023-404629/C202SC18110931_Caenorhabditis_elegans_Primary_Report/src/js/fancybox/fancy_shadow_se.png]

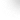

Supplement: jkad258_Supplementary_Data [file jkad258_supplementary_data.zip › suppl_data/Supplemental_Methods_G3-2023-404629/C202SC18110931_Caenorhabditis_elegans_Primary_Report/src/js/fancybox/fancy_shadow_sw.png]

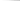

Supplement: jkad258_Supplementary_Data [file jkad258_supplementary_data.zip › suppl_data/Supplemental_Methods_G3-2023-404629/C202SC18110931_Caenorhabditis_elegans_Primary_Report/src/js/fancybox/fancy_shadow_w.png]

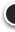

Supplement: jkad258_Supplementary_Data [file jkad258_supplementary_data.zip › suppl_data/Supplemental_Methods_G3-2023-404629/C202SC18110931_Caenorhabditis_elegans_Primary_Report/src/js/fancybox/fancy_title_left.png]

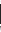

Supplement: jkad258_Supplementary_Data [file jkad258_supplementary_data.zip › suppl_data/Supplemental_Methods_G3-2023-404629/C202SC18110931_Caenorhabditis_elegans_Primary_Report/src/js/fancybox/fancy_title_main.png]

Supplement: jkad258_Supplementary_Data [file jkad258_supplementary_data.zip › suppl_data/Supplemental_Methods_G3-2023-404629/C202SC18110931_Caenorhabditis_elegans_Primary_Report/src/js/fancybox/fancy_title_over.png]

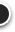

Supplement: jkad258_Supplementary_Data [file jkad258_supplementary_data.zip › suppl_data/Supplemental_Methods_G3-2023-404629/C202SC18110931_Caenorhabditis_elegans_Primary_Report/src/js/fancybox/fancy_title_right.png]

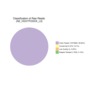

Supplement: jkad258_Supplementary_Data [file jkad258_supplementary_data.zip › suppl_data/Supplemental_Methods_G3-2023-404629/C202SC18110931_Caenorhabditis_elegans_Primary_Report/src/pictures/Class/N2_HGVYFDSXX_L3.pie3d.JPEG]

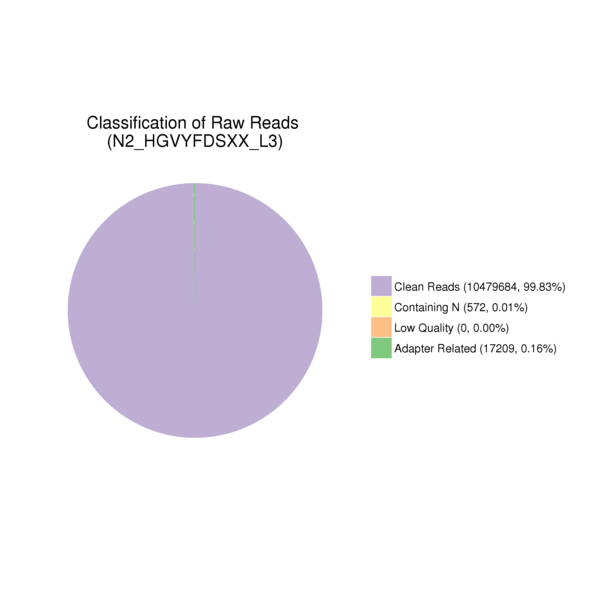

Supplement: jkad258_Supplementary_Data [file jkad258_supplementary_data.zip › suppl_data/Supplemental_Methods_G3-2023-404629/C202SC18110931_Caenorhabditis_elegans_Primary_Report/src/pictures/Class/N2_HGVYFDSXX_L3.pie3d.png]

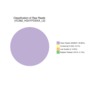

Supplement: jkad258_Supplementary_Data [file jkad258_supplementary_data.zip › suppl_data/Supplemental_Methods_G3-2023-404629/C202SC18110931_Caenorhabditis_elegans_Primary_Report/src/pictures/Class/VC362_HGVYFDSXX_L3.pie3d.JPEG]

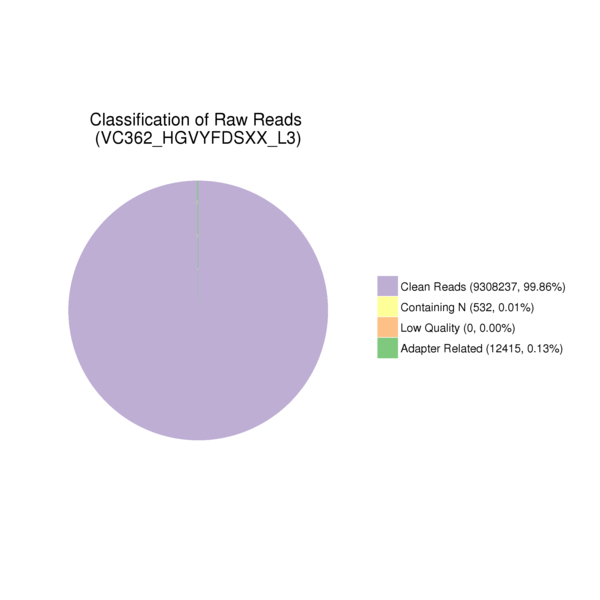

Supplement: jkad258_Supplementary_Data [file jkad258_supplementary_data.zip › suppl_data/Supplemental_Methods_G3-2023-404629/C202SC18110931_Caenorhabditis_elegans_Primary_Report/src/pictures/Class/VC362_HGVYFDSXX_L3.pie3d.png]

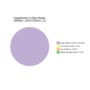

Supplement: jkad258_Supplementary_Data [file jkad258_supplementary_data.zip › suppl_data/Supplemental_Methods_G3-2023-404629/C202SC18110931_Caenorhabditis_elegans_Primary_Report/src/pictures/Class/WRM31_HGVYFDSXX_L3.pie3d.JPEG]

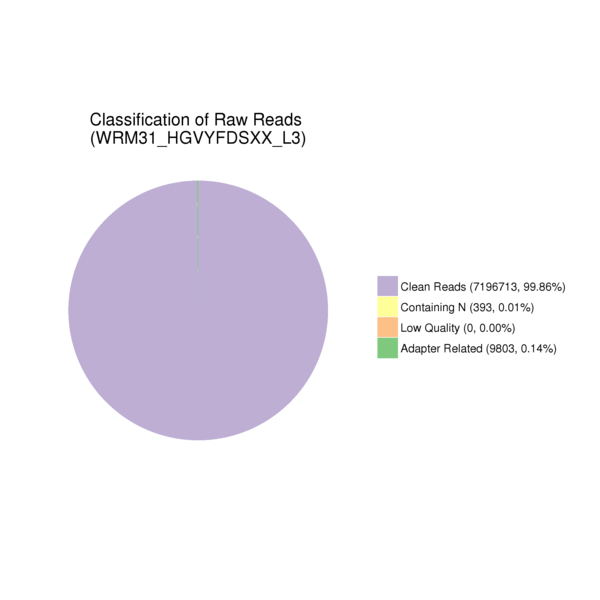

Supplement: jkad258_Supplementary_Data [file jkad258_supplementary_data.zip › suppl_data/Supplemental_Methods_G3-2023-404629/C202SC18110931_Caenorhabditis_elegans_Primary_Report/src/pictures/Class/WRM31_HGVYFDSXX_L3.pie3d.png]

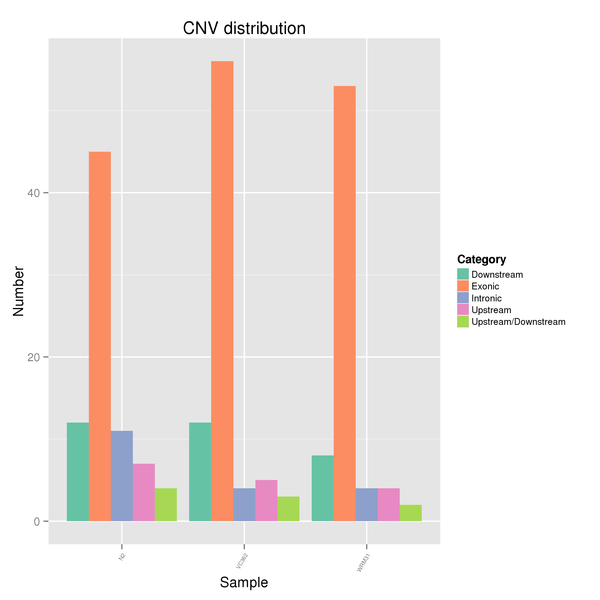

Supplement: jkad258_Supplementary_Data [file jkad258_supplementary_data.zip › suppl_data/Supplemental_Methods_G3-2023-404629/C202SC18110931_Caenorhabditis_elegans_Primary_Report/src/pictures/CNV/CNV_Annotation_Statistics.png]

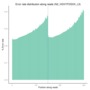

Supplement: jkad258_Supplementary_Data [file jkad258_supplementary_data.zip › suppl_data/Supplemental_Methods_G3-2023-404629/C202SC18110931_Caenorhabditis_elegans_Primary_Report/src/pictures/Error/N2_HGVYFDSXX_L3.Error.JPEG]

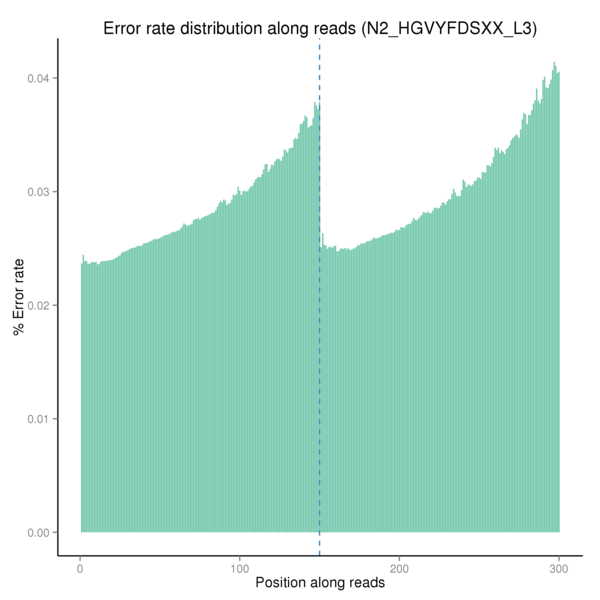

Supplement: jkad258_Supplementary_Data [file jkad258_supplementary_data.zip › suppl_data/Supplemental_Methods_G3-2023-404629/C202SC18110931_Caenorhabditis_elegans_Primary_Report/src/pictures/Error/N2_HGVYFDSXX_L3.Error.png]

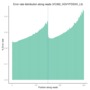

Supplement: jkad258_Supplementary_Data [file jkad258_supplementary_data.zip › suppl_data/Supplemental_Methods_G3-2023-404629/C202SC18110931_Caenorhabditis_elegans_Primary_Report/src/pictures/Error/VC362_HGVYFDSXX_L3.Error.JPEG]

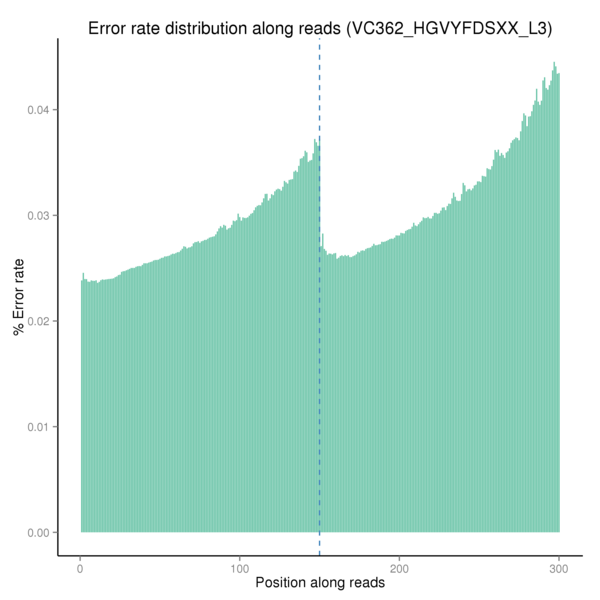

Supplement: jkad258_Supplementary_Data [file jkad258_supplementary_data.zip › suppl_data/Supplemental_Methods_G3-2023-404629/C202SC18110931_Caenorhabditis_elegans_Primary_Report/src/pictures/Error/VC362_HGVYFDSXX_L3.Error.png]

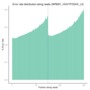

Supplement: jkad258_Supplementary_Data [file jkad258_supplementary_data.zip › suppl_data/Supplemental_Methods_G3-2023-404629/C202SC18110931_Caenorhabditis_elegans_Primary_Report/src/pictures/Error/WRM31_HGVYFDSXX_L3.Error.JPEG]

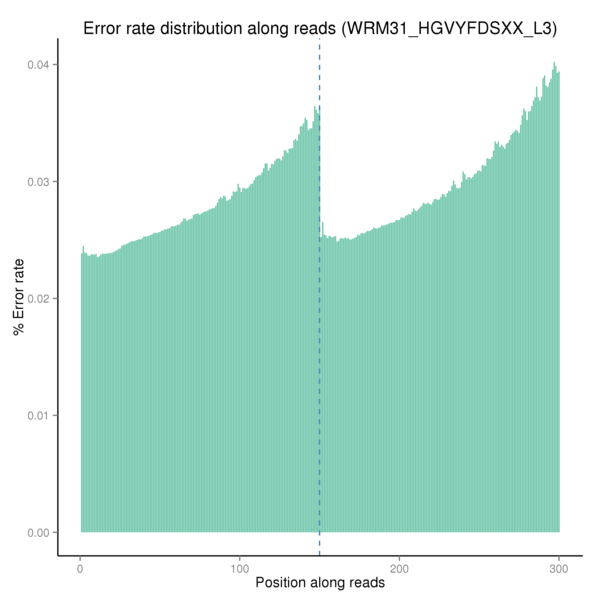

Supplement: jkad258_Supplementary_Data [file jkad258_supplementary_data.zip › suppl_data/Supplemental_Methods_G3-2023-404629/C202SC18110931_Caenorhabditis_elegans_Primary_Report/src/pictures/Error/WRM31_HGVYFDSXX_L3.Error.png]

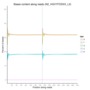

Supplement: jkad258_Supplementary_Data [file jkad258_supplementary_data.zip › suppl_data/Supplemental_Methods_G3-2023-404629/C202SC18110931_Caenorhabditis_elegans_Primary_Report/src/pictures/GC/N2_HGVYFDSXX_L3.GC.JPEG]

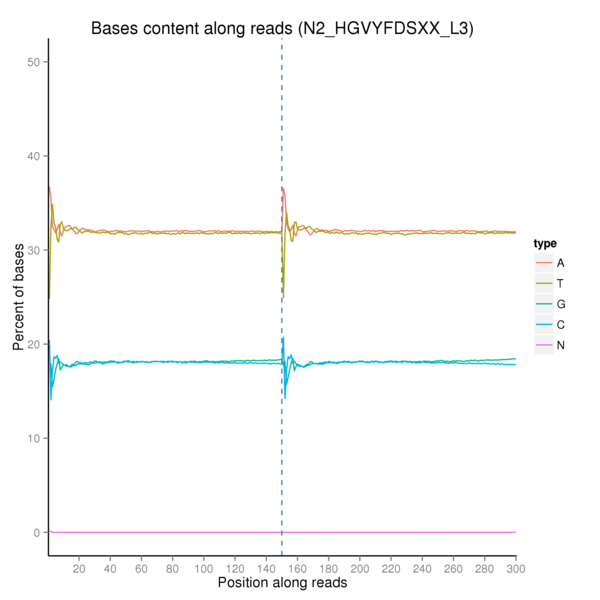

Supplement: jkad258_Supplementary_Data [file jkad258_supplementary_data.zip › suppl_data/Supplemental_Methods_G3-2023-404629/C202SC18110931_Caenorhabditis_elegans_Primary_Report/src/pictures/GC/N2_HGVYFDSXX_L3.GC.png]

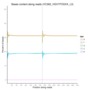

Supplement: jkad258_Supplementary_Data [file jkad258_supplementary_data.zip › suppl_data/Supplemental_Methods_G3-2023-404629/C202SC18110931_Caenorhabditis_elegans_Primary_Report/src/pictures/GC/VC362_HGVYFDSXX_L3.GC.JPEG]

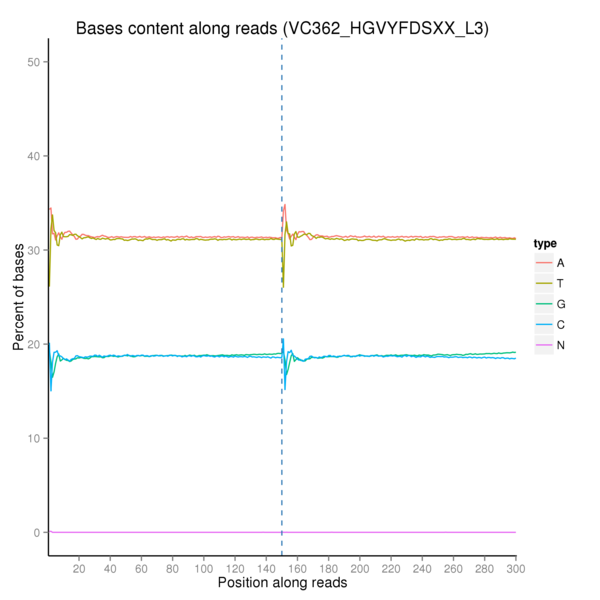

Supplement: jkad258_Supplementary_Data [file jkad258_supplementary_data.zip › suppl_data/Supplemental_Methods_G3-2023-404629/C202SC18110931_Caenorhabditis_elegans_Primary_Report/src/pictures/GC/VC362_HGVYFDSXX_L3.GC.png]

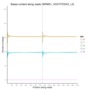

Supplement: jkad258_Supplementary_Data [file jkad258_supplementary_data.zip › suppl_data/Supplemental_Methods_G3-2023-404629/C202SC18110931_Caenorhabditis_elegans_Primary_Report/src/pictures/GC/WRM31_HGVYFDSXX_L3.GC.JPEG]

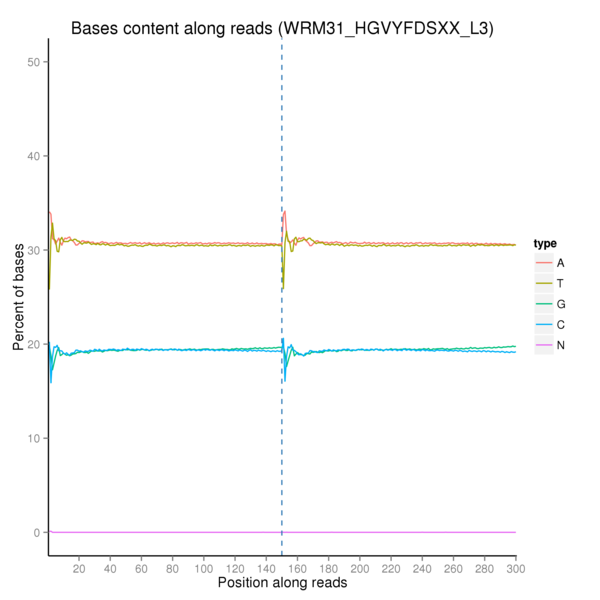

Supplement: jkad258_Supplementary_Data [file jkad258_supplementary_data.zip › suppl_data/Supplemental_Methods_G3-2023-404629/C202SC18110931_Caenorhabditis_elegans_Primary_Report/src/pictures/GC/WRM31_HGVYFDSXX_L3.GC.png]

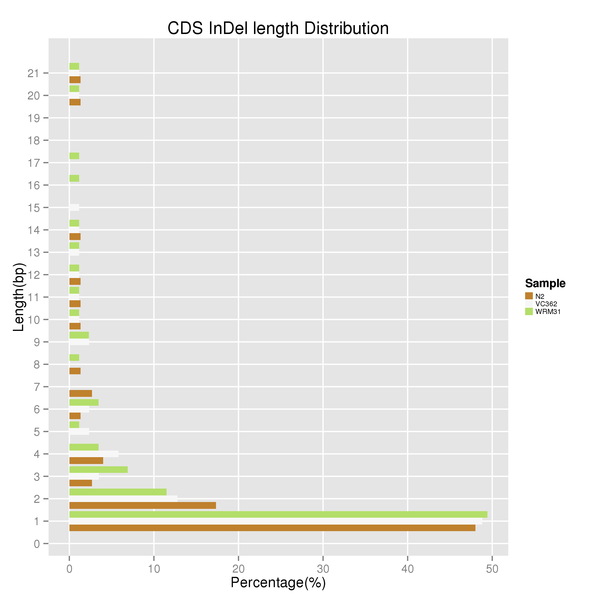

Supplement: jkad258_Supplementary_Data [file jkad258_supplementary_data.zip › suppl_data/Supplemental_Methods_G3-2023-404629/C202SC18110931_Caenorhabditis_elegans_Primary_Report/src/pictures/InDel/InDel_CDSpercentage.png]

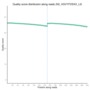

Supplement: jkad258_Supplementary_Data [file jkad258_supplementary_data.zip › suppl_data/Supplemental_Methods_G3-2023-404629/C202SC18110931_Caenorhabditis_elegans_Primary_Report/src/pictures/Quality/N2_HGVYFDSXX_L3.QM.JPEG]

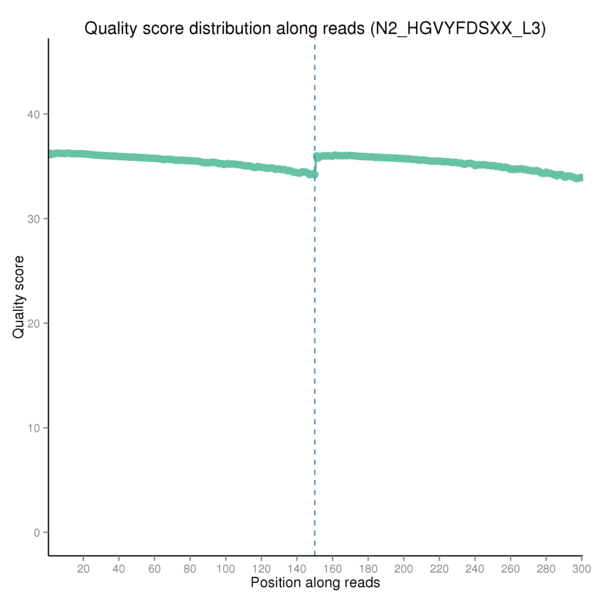

Supplement: jkad258_Supplementary_Data [file jkad258_supplementary_data.zip › suppl_data/Supplemental_Methods_G3-2023-404629/C202SC18110931_Caenorhabditis_elegans_Primary_Report/src/pictures/Quality/N2_HGVYFDSXX_L3.QM.png]

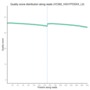

Supplement: jkad258_Supplementary_Data [file jkad258_supplementary_data.zip › suppl_data/Supplemental_Methods_G3-2023-404629/C202SC18110931_Caenorhabditis_elegans_Primary_Report/src/pictures/Quality/VC362_HGVYFDSXX_L3.QM.JPEG]

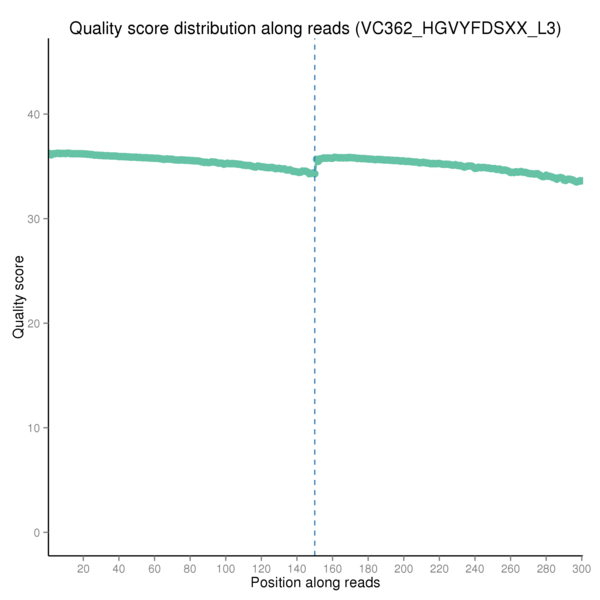

Supplement: jkad258_Supplementary_Data [file jkad258_supplementary_data.zip › suppl_data/Supplemental_Methods_G3-2023-404629/C202SC18110931_Caenorhabditis_elegans_Primary_Report/src/pictures/Quality/VC362_HGVYFDSXX_L3.QM.png]

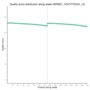

Supplement: jkad258_Supplementary_Data [file jkad258_supplementary_data.zip › suppl_data/Supplemental_Methods_G3-2023-404629/C202SC18110931_Caenorhabditis_elegans_Primary_Report/src/pictures/Quality/WRM31_HGVYFDSXX_L3.QM.JPEG]

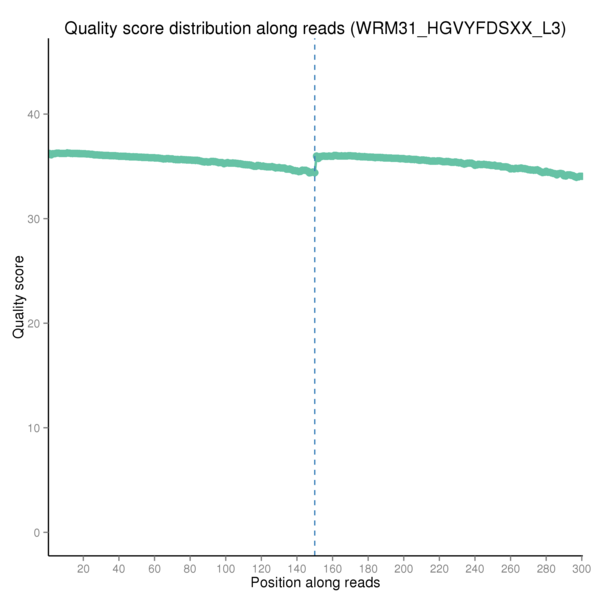

Supplement: jkad258_Supplementary_Data [file jkad258_supplementary_data.zip › suppl_data/Supplemental_Methods_G3-2023-404629/C202SC18110931_Caenorhabditis_elegans_Primary_Report/src/pictures/Quality/WRM31_HGVYFDSXX_L3.QM.png]

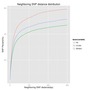

Supplement: jkad258_Supplementary_Data [file jkad258_supplementary_data.zip › suppl_data/Supplemental_Methods_G3-2023-404629/C202SC18110931_Caenorhabditis_elegans_Primary_Report/src/pictures/SNP/SNP_distance_cumulative_distribution.JPEG]

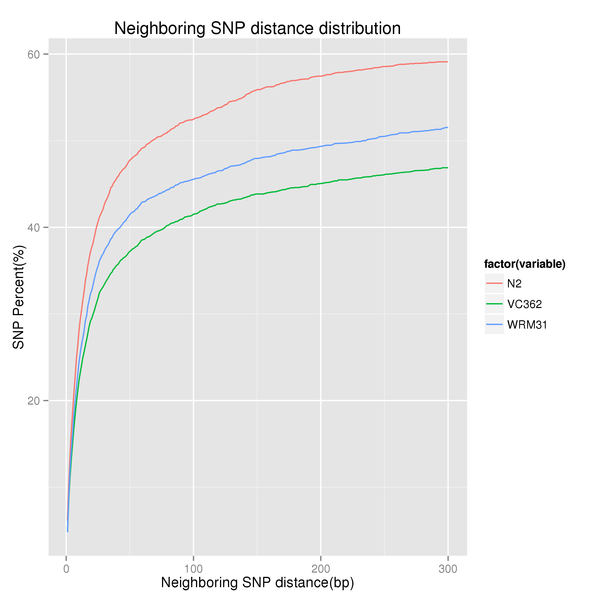

Supplement: jkad258_Supplementary_Data [file jkad258_supplementary_data.zip › suppl_data/Supplemental_Methods_G3-2023-404629/C202SC18110931_Caenorhabditis_elegans_Primary_Report/src/pictures/SNP/SNP_distance_cumulative_distribution.png]

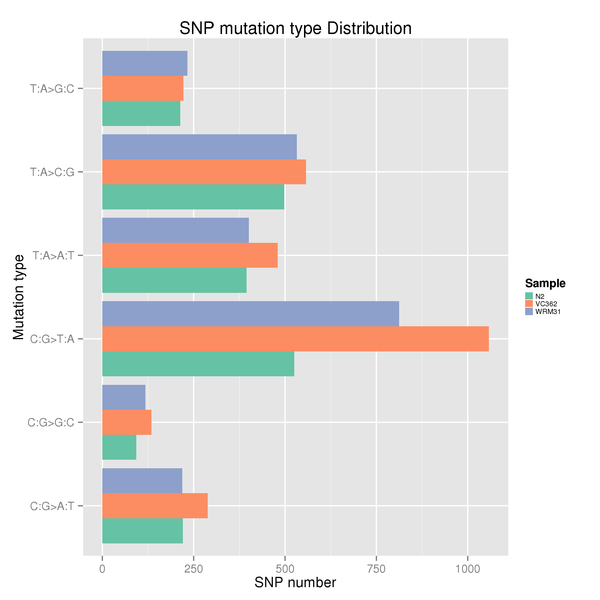

Supplement: jkad258_Supplementary_Data [file jkad258_supplementary_data.zip › suppl_data/Supplemental_Methods_G3-2023-404629/C202SC18110931_Caenorhabditis_elegans_Primary_Report/src/pictures/SNP/SNP_frequency.png]

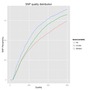

Supplement: jkad258_Supplementary_Data [file jkad258_supplementary_data.zip › suppl_data/Supplemental_Methods_G3-2023-404629/C202SC18110931_Caenorhabditis_elegans_Primary_Report/src/pictures/SNP/SNP_quality_cumulative_distribution.JPEG]

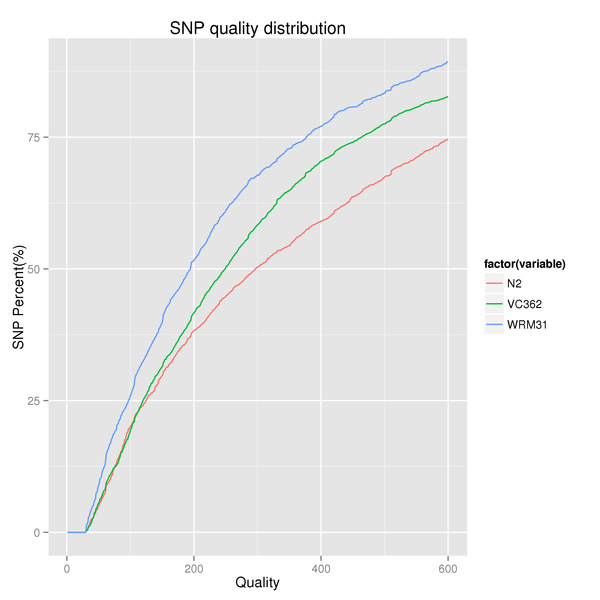

Supplement: jkad258_Supplementary_Data [file jkad258_supplementary_data.zip › suppl_data/Supplemental_Methods_G3-2023-404629/C202SC18110931_Caenorhabditis_elegans_Primary_Report/src/pictures/SNP/SNP_quality_cumulative_distribution.png]

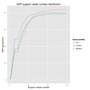

Supplement: jkad258_Supplementary_Data [file jkad258_supplementary_data.zip › suppl_data/Supplemental_Methods_G3-2023-404629/C202SC18110931_Caenorhabditis_elegans_Primary_Report/src/pictures/SNP/SNP_readsNum_cumulative_distribution.JPEG]

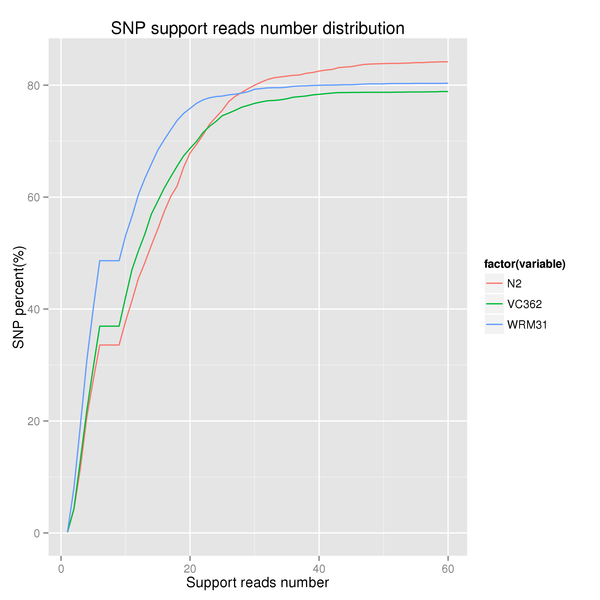

Supplement: jkad258_Supplementary_Data [file jkad258_supplementary_data.zip › suppl_data/Supplemental_Methods_G3-2023-404629/C202SC18110931_Caenorhabditis_elegans_Primary_Report/src/pictures/SNP/SNP_readsNum_cumulative_distribution.png]

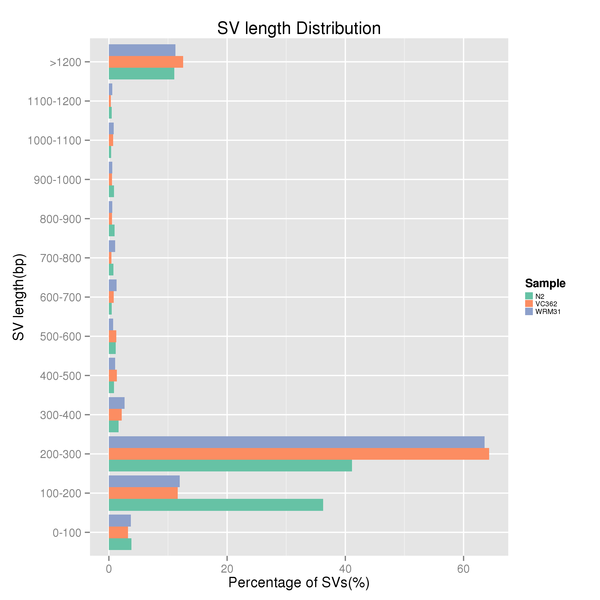

Supplement: jkad258_Supplementary_Data [file jkad258_supplementary_data.zip › suppl_data/Supplemental_Methods_G3-2023-404629/C202SC18110931_Caenorhabditis_elegans_Primary_Report/src/pictures/SV/SV_length_distribution.png]

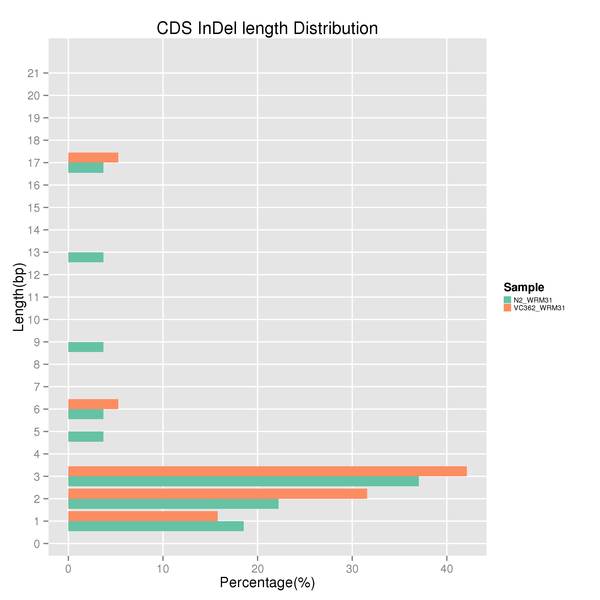

Supplement: jkad258_Supplementary_Data [file jkad258_supplementary_data.zip › suppl_data/Supplemental_Methods_G3-2023-404629/C202SC18110931_Caenorhabditis_elegans_Primary_Report/src_somatic/pictures/InDel/InDel.CDSpercentage.png]

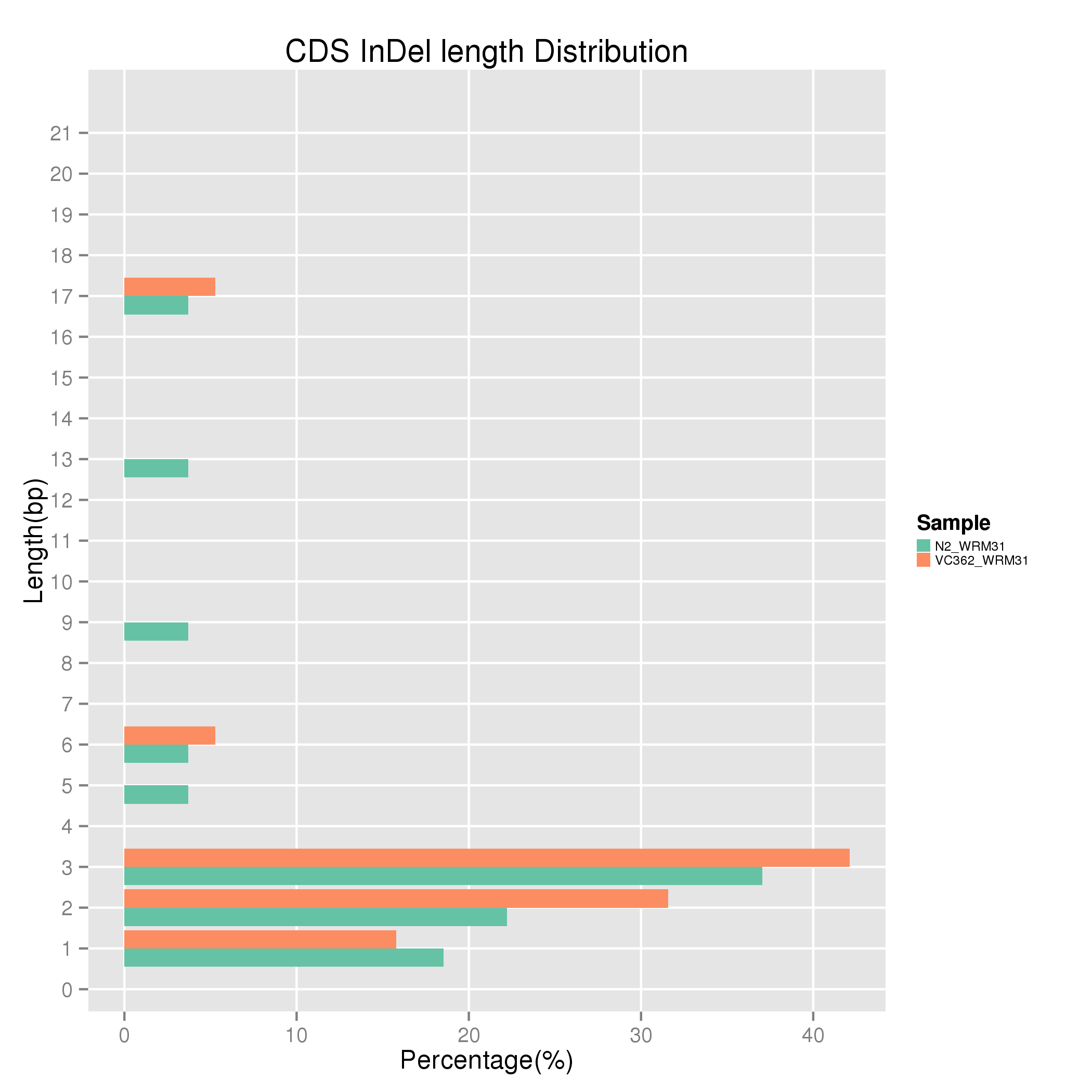

Supplement: jkad258_Supplementary_Data [file jkad258_supplementary_data.zip › suppl_data/Supplemental_Methods_G3-2023-404629/C202SC18110931_Caenorhabditis_elegans_Primary_Report/src_somatic/pictures/InDel/InDel.CDSpercentage.xls.png]

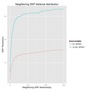

Supplement: jkad258_Supplementary_Data [file jkad258_supplementary_data.zip › suppl_data/Supplemental_Methods_G3-2023-404629/C202SC18110931_Caenorhabditis_elegans_Primary_Report/src_somatic/pictures/SNP/SNP.distance.JPEG]

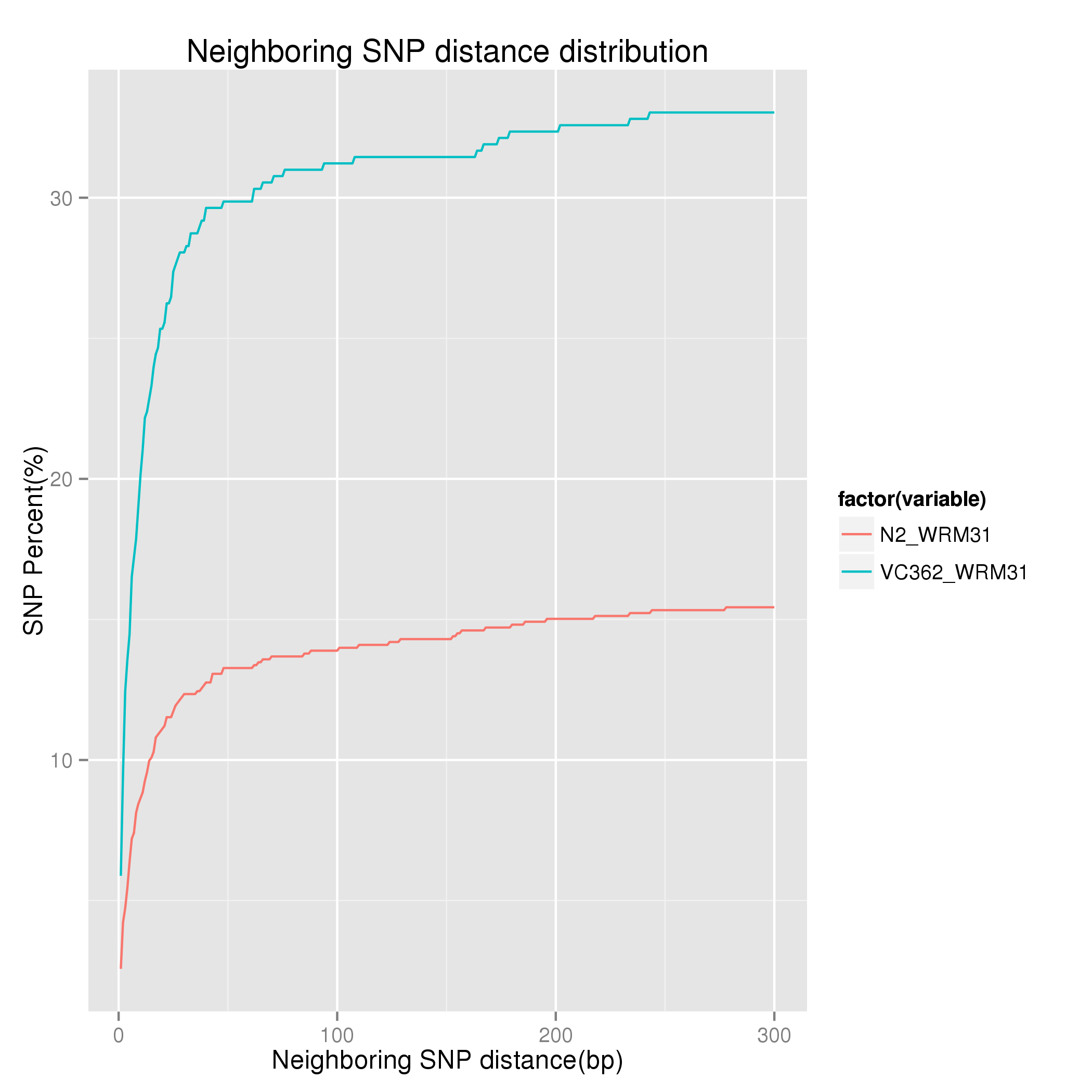

Supplement: jkad258_Supplementary_Data [file jkad258_supplementary_data.zip › suppl_data/Supplemental_Methods_G3-2023-404629/C202SC18110931_Caenorhabditis_elegans_Primary_Report/src_somatic/pictures/SNP/SNP.distance.png]

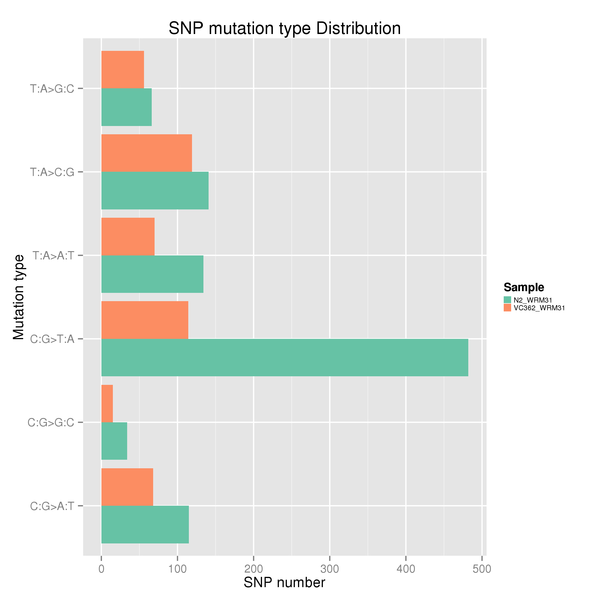

Supplement: jkad258_Supplementary_Data [file jkad258_supplementary_data.zip › suppl_data/Supplemental_Methods_G3-2023-404629/C202SC18110931_Caenorhabditis_elegans_Primary_Report/src_somatic/pictures/SNP/SNP.frequency.png]

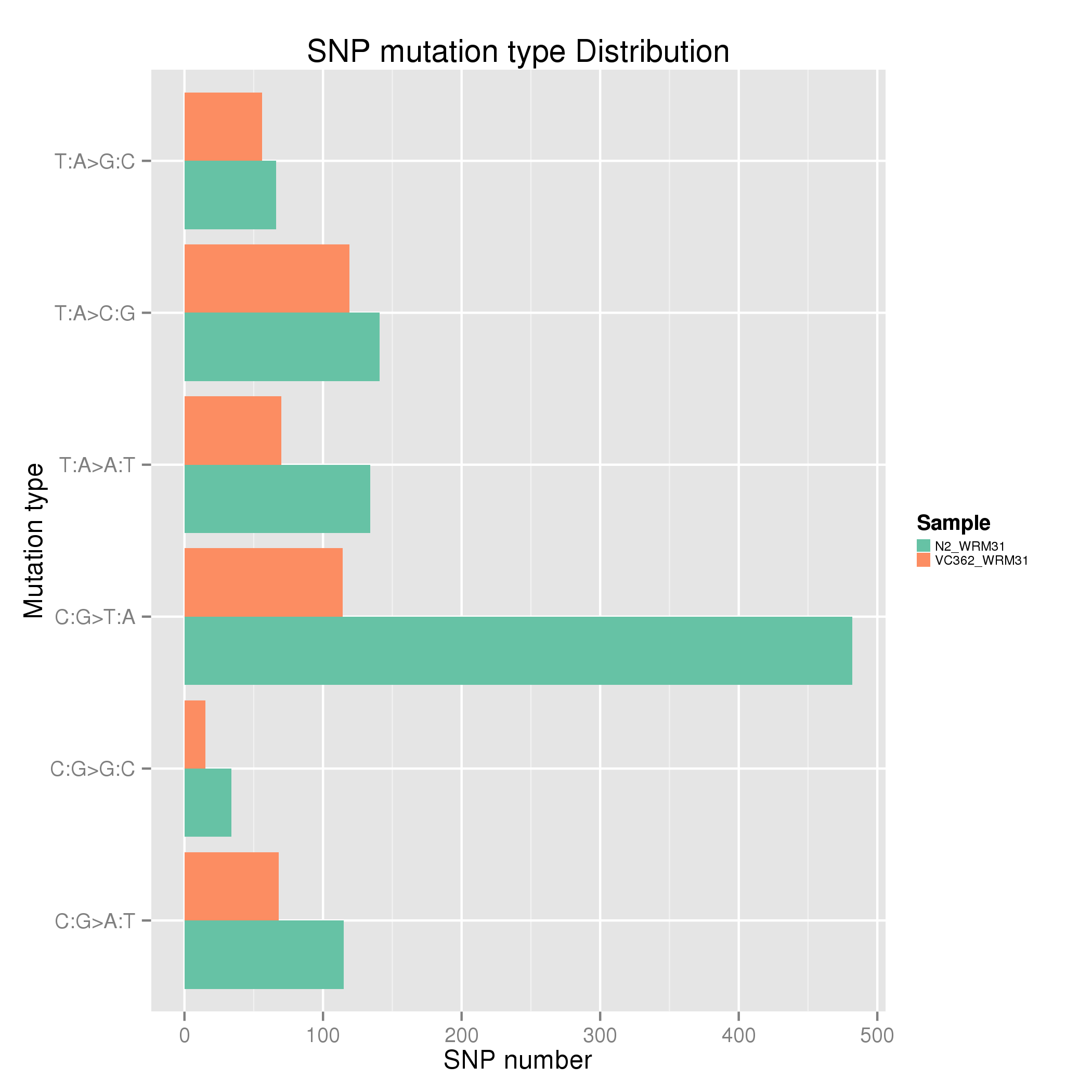

Supplement: jkad258_Supplementary_Data [file jkad258_supplementary_data.zip › suppl_data/Supplemental_Methods_G3-2023-404629/C202SC18110931_Caenorhabditis_elegans_Primary_Report/src_somatic/pictures/SNP/SNP.frequency.xls.png]

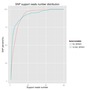

Supplement: jkad258_Supplementary_Data [file jkad258_supplementary_data.zip › suppl_data/Supplemental_Methods_G3-2023-404629/C202SC18110931_Caenorhabditis_elegans_Primary_Report/src_somatic/pictures/SNP/SNP.readsNum.JPEG]

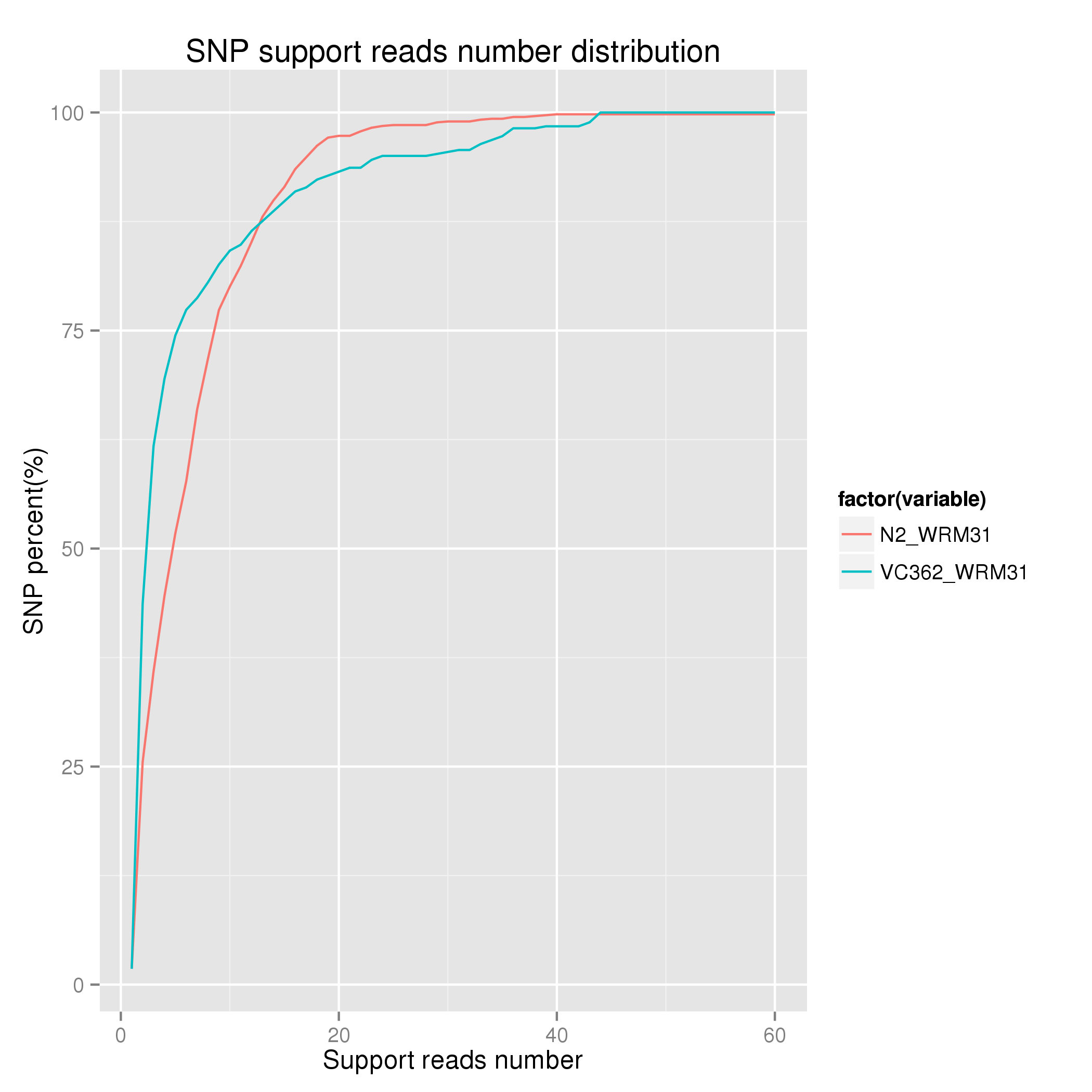

Supplement: jkad258_Supplementary_Data [file jkad258_supplementary_data.zip › suppl_data/Supplemental_Methods_G3-2023-404629/C202SC18110931_Caenorhabditis_elegans_Primary_Report/src_somatic/pictures/SNP/SNP.readsNum.png]
